# Transcriptional pathways across colony biofilm models in the symbiont *Vibrio fischeri*

**DOI:** 10.1128/msystems.00815-23

**Published:** 2023-12-21

**Authors:** Jacob A. Vander Griend, Ruth Y. Isenberg, Ketan R. Kotla, Mark J. Mandel

**Affiliations:** 1 Department of Medical Microbiology and Immunology, University of Wisconsin-Madison, Madison, Wisconsin, USA; 2 Microbiology Doctoral Training Program, University of Wisconsin-Madison, Madison, Wisconsin, USA; Pennsylvania State University, University Park, Pennsylvania, USA

**Keywords:** bacterial biofilm, symbiosis, regulatory pathways, phosphorelay, two-component signaling systems, comparative transcriptomics

## Abstract

Beneficial microbial symbionts that are horizontally acquired by their animal hosts undergo a lifestyle transition from free-living in the environment to associating with host tissues. In the model symbiosis between the Hawaiian bobtail squid and its microbial symbiont *Vibrio fischeri*, one mechanism used to make this transition during host colonization is the formation of biofilm-like aggregates in host mucosa. Previous work identified factors that are sufficient to induce *V. fischeri* biofilm formation, yet much remains unknown regarding the breadth of target genes induced by these factors. Here, we probed two widely used *in vitro* models of biofilm formation to identify novel regulatory pathways in the squid symbiont *V. fischeri* ES114. We discovered a shared set of 232 genes that demonstrated similar patterns in expression in both models. These genes comprise multiple exopolysaccharide loci that are upregulated and flagellar motility genes that are downregulated, with a consistent decrease in measured swimming motility. Furthermore, we identified genes regulated downstream of the key sensor kinase RscS that are induced independent of the response regulator SypG. Our data suggest that transcriptional regulator VpsR plays a strong role in expression of at least a subset of these genes. Overall, this study adds to our understanding of the genes involved in *V. fischeri* biofilm regulation while revealing new regulatory pathways branching from previously characterized signaling networks.

**IMPORTANCE:**

The *V. fischeri-*squid system provides an opportunity to study biofilm development both in the animal host and in culture-based biofilm models that capture key aspects of *in vivo* signaling. In this work, we report the results of the transcriptomic profiling of two *V. fischeri* biofilm models followed by phenotypic validation and examination of novel signaling pathway architecture. Remarkable consistency between the models provides a strong basis for future studies using either approach or both. A subset of the factors identified by the approaches were validated in the work, and the body of transcriptomic data provides a number of leads for future studies in culture and during animal colonization.

## INTRODUCTION

In animal-microbe mutualisms, symbionts can provide nutritional, developmental, and/or defensive benefits to the host, and in turn, the symbionts may gain various benefits from association with the host (1–6). During horizontal transmission, hosts must reacquire their symbionts each generation from environmental symbiont populations (7–14). Unfortunately, the understanding of how specific microbes make this transition from environment to host is often hindered by the complexity of animal microbiomes (15, 16). Symbioses with limited symbiont diversity are therefore valuable as models to identify mechanisms of colonization and transmission. The binary mutualism between the nocturnal Hawaiian bobtail squid (*Euprymna scolopes*) and the bioluminescent marine bacterium *Vibrio fischeri* is one such model system that integrates symbiont specificity, a defined colonization program, and a genetically tractable microbe (14, 17, 18).

Upon hatching from aposymbiotic (symbiont-free) eggs, juvenile squid recruit symbionts from the surrounding seawater (18, 19). Located in the squid’s mantle cavity, the bilobed symbiotic “light organ” actively captures bacteria from seawater through the activity of extruded ciliated appendages on each lobe, which focus water currents onto a mucosal layer on the exterior of the organ (20). *V. fischeri* cells that become entrained in host mucosa form biofilm-like aggregates through the secretion of a specific exopolysaccharide (13, 21, 22). Aggregate formation is a critical step in host colonization, and mutants defective in aggregation are generally compromised in reaching the internal crypt spaces of the light organ where the symbiosis is maintained (22, 23). Therefore, understanding the genetic program connected to *in vivo* biofilm formation is critical to understand the transition that the colonizing microbes undergo from the planktonic state in seawater to successfully engraft themselves into the host microbiome.

**Fig 1:**
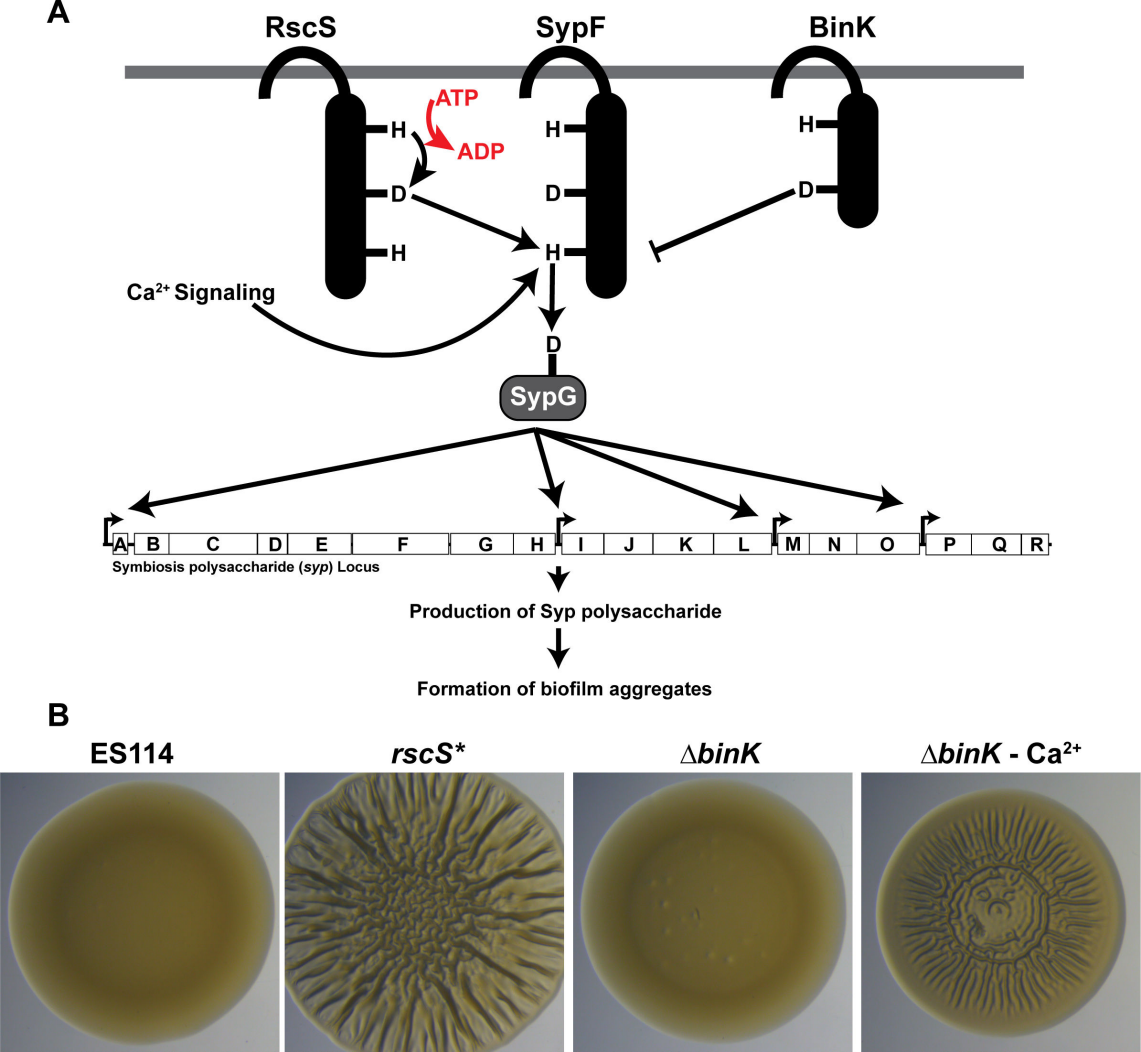
*Vibrio fischeri* symbiosis polysaccharide regulation. (A) *V. fischeri* induces biofilm formation through a phosphorelay initiated at the hybrid sensor kinase RscS, flowing through the hybrid sensor kinase SypF to the response regulator SypG. Growth on calcium media induces a secondary pathway also flowing through SypF. In opposition to this pathway, the sensor kinase BinK inhibits the activation of SypG, preventing biofilm formation. (B) *V. fischeri* ES114 has a smooth colony morphology when grown on Luria-Bertani salt (LBS) agar. Overexpression of the sensor kinase RscS (rscS*) or growth of a Δ*binK* mutant on LBS 10 mM Ca^2+^ agar permits the formation of “wrinkled” colonies with a rugose morphology due to production of the Syp exopolysaccharide.

*V. fischeri* aggregates have been shown to require production of the symbiosis exopolysaccharide (Syp), produced and exported by the products of a conserved 18-gene (*syp*) locus (21, 22, 24, 25). Control of the *syp* locus is principally coordinated through a phosphorelay network feeding into two downstream response regulators (21, 23, 26) (Fig. 1A). Following autophosphorylation of the hybrid sensor kinase RscS, the phosphoryl group is transferred to the hybrid sensor kinase SypF (21, 27). Upon phosphorylation of SypF, downstream phosphotransfer events are targeted from SypF onto the response regulators SypE and SypG (27). SypG is a σ^54^-dependent activator that, when phosphorylated in its receiver domain, binds four promoters within the *syp* locus and activates their transcription (28). In contrast to the DNA-binding functionality of SypG, SypE is believed to act as a post-transcriptional regulator of Syp production that acts through SypA (29). As an additional level of control, *V. fischeri* also inhibits expression of the *syp* locus through the action of the biofilm inhibitor sensor kinase (BinK), which acts through SypG (30, 31).

Despite the robust induction of Syp exopolysaccharide production during host colonization, *in vitro* (i.e., culture-based) models of *V. fischeri* require genetic manipulation or chemical supplementation to induce biofilm formation (23, 32–34). One method to induce biofilm is via overexpression of the sensor kinase RscS, either through the plasmid-based *rscS1* allele or the chromosomal rscS* allele (21, 35, 36). RscS overexpression also induces increased aggregate formation in the squid host, explicitly linking the ability of this model to form biofilms *in vitro* with aggregation in the host context (21). Also, deletion of the gene encoding inhibitor sensor kinase BinK was also found to induce significantly larger aggregates during colonization (30). In culture, this biofilm-up phenotype can be reproduced by treating a Δ*binK* mutant with levels of calcium comparable to those found in seawater (Δ*binK-*Ca^2+^), which similarly results in wrinkled colony biofilm formation (33). Both of the culture-based rscS* and Δ*binK*-Ca^2+^ models increase biofilm formation by stimulating the expression of the *syp* locus through the sensor kinase SypF (27, 33). However, the models differ in the input by which SypF is phosphorylated, with RscS acting as the primary phosphodonor to SypF in the rscS* model, while the Δ*binK-*Ca^2+^ model requires a secondary phosphorelay involving the sensor kinase HahK (27, 33).

The discovery of both the RscS overexpression and Δ*binK*-Ca^2+^ models opened the door to genetic analysis of Syp regulation, as both models recapitulate the induction of the critical Syp exopolysaccharide without requiring the squid host (27, 30, 33, 37). Induction of the Syp exopolysaccharide in culture manifests as a distinct wrinkling morphology within colonies grown on agar media, while the wild-type strain of *V. fischeri* ES114 remains smooth (Fig. 1B) (24, 33). Considering the shared dependence of both wrinkled colony biofilm models and *in vivo* aggregates on Syp exopolysaccharide production, in this work, we utilized wrinkled colonies formed by both of the biofilm models as proxies for *in vivo* aggregates in a comparative transcriptomics assay.

## RESULTS

### Definition of a core biofilm regulon for *V. fischeri* ES114

*V. fischeri* wrinkled colony biofilms are a close proxy for the aggregates that form *in vivo* during squid host colonization (21, 22). To begin to understand the genes that are induced under these conditions, we asked what patterns of gene expression were shared in two common wrinkled colony biofilm models: (i) genetic induction with an *rscS* overexpression allele (rscS*) in an otherwise wild-type background compared to wild-type *V. fischeri* ES114 and (ii) chemical induction with 10 mM CaCl_2_ in a Δ*binK* background (Δ*binK*-Ca^2+^) compared to Δ*binK* alone (33, 36) (Fig. 1B). We selected these comparisons to explicitly compare biofilm induced to the closest uninduced control conditions for each. To query gene expression in wrinkled colony biofilms, we compared the global transcriptome in the induced condition with the uninduced condition in each case. Spots of overnight culture were spotted onto fresh agar and incubated at 25°C to form wrinkled colony biofilms. After 48 hours, each spot was scraped, and RNA was isolated for differential expression analysis. Throughout the study, we used thresholds of twofold induction/repression and false discovery rate (FDR) of 0.05.

#### Effects on exopolysaccharide gene transcription

**Fig 2:**
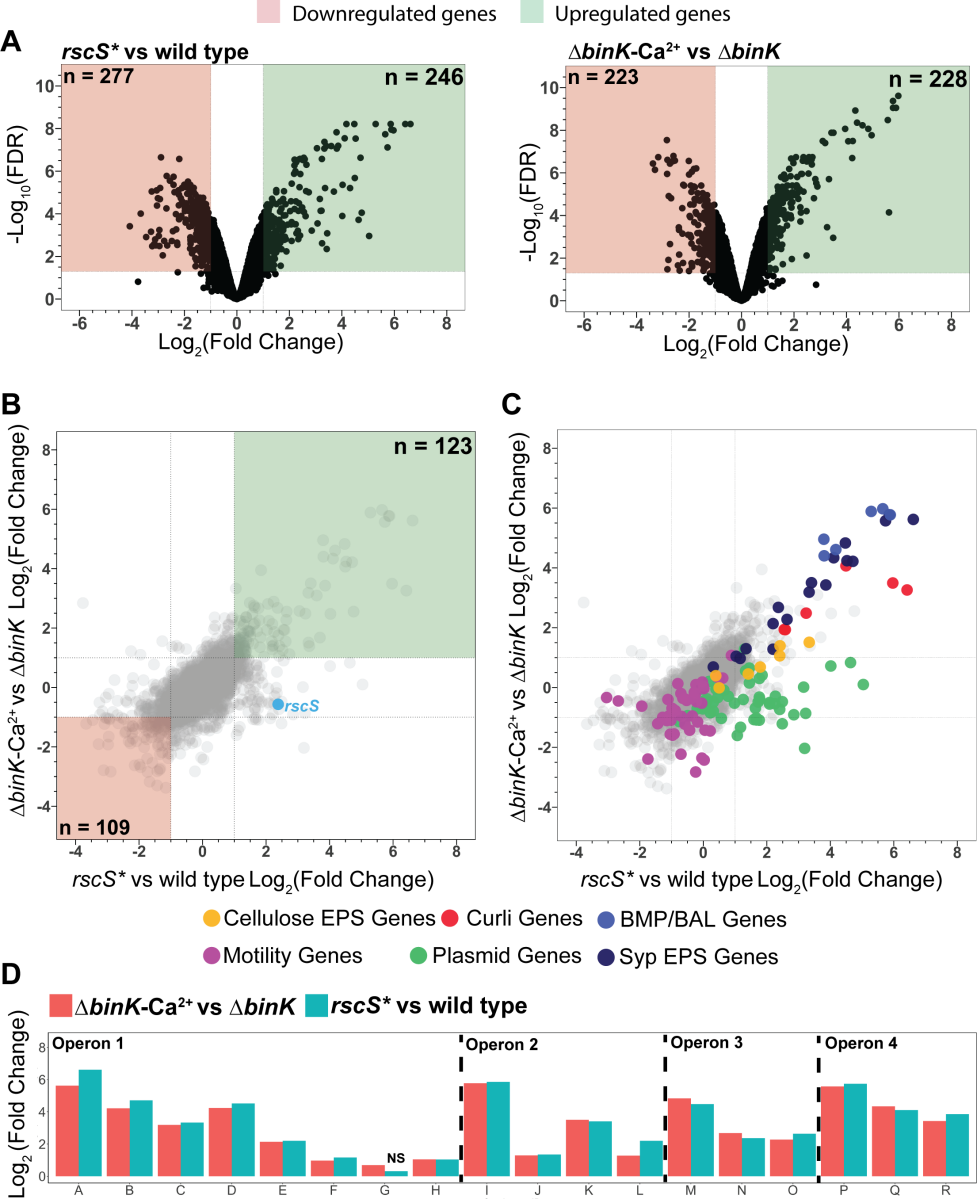
*Vibrio fischeri* induces a core transcriptomic response during biofilm formation. (A) Volcano plots of the rscS* vs wild type and Δ*binK*-Ca^2+^ vs Δ*binK* biofilm models. Green and red boxes indicate those genes with log_2_(fold change) ≥1 and ≤−1, respectively, with significant differential expression (FDR < 0.05). (B) Overlay of log_2_(fold change) values for all genes from both the rscS* vs wild type and Δ*binK*-Ca^2+^ vs Δ*binK* biofilm models. Green and red boxes are analogous to those in panel A. (C) Overlay of annotations onto the multi-comparison overlay shown in panel B; dot legend is provided below. (D) Syp locus differential expression. Log_2_(fold change) for each of the 18 genes in the *syp* locus is provided from the two differential expression analyses. Operon indications are provided from previous analyses of SypG-bound promoters (28). For the one gene which had no significant changes in expression (sypG, rscS* vs wild type), NS is indicated above that column.

In both biofilm models, we detected a large number of genes that were differentially expressed, with over 200 genes upregulated and over 200 genes downregulated upon biofilm induction (Fig. 2A). Comparing the results from the two models revealed 123 genes that were significantly upregulated and 109 genes that were significantly downregulated in both cases (Fig. 2B). The most highly upregulated genes included those in the *Syp* locus, which are required for aggregation in the host and are known to be regulated downstream of RscS and Δ*binK*-Ca^2+^ signaling (33) (Fig. 2C). Examination of the data revealed a remarkably consistent response for the *syp* genes across both models. The *syp* locus is organized into four operons, each of which has a σ^54^ promoter and binding sites for the enhancer-binding protein SypG to facilitate transcription by Eσ^54^ (28). We noted a trend in which the first gene in each operon was the most highly induced, with *sypA* being the highest induced gene across the entire locus with over 97-fold induction in the rscS* background and 49-fold induction in the Δ*binK*-Ca^2+^ background (Fig. 2D). Proceeding along each operon, the fold induction generally diminished for subsequent genes. Again, responses under the two biofilm models were highly correlated. The three SypG-regulated biofilm maturation (matrix) proteins and three biofilm-associated lipoproteins are similarly regulated by both models (Fig. 2C) (38). These data therefore illustrate that our RNA-seq analysis from agar-grown colony spots captures relevant transcriptional responses and that integration of both models yields valuable signals. In the remainder of the study, we refer to genes that show consistent regulation under both models as the “core biofilm regulon.”

One unexpected result was that *sypG* had the lowest biofilm induction among the 18 *syp* genes. Given the importance of SypG to biofilm regulation as the σ^54^-dependent activator for transcription of the *syp* promoters, we were curious to investigate the relative transcript levels of *sypG* within a sample, especially in the uninduced conditions. We conducted an analysis of gene expression within each condition using transcripts per kilobase million reads (TPMs) and focused on the genes within the *syp* locus (39). This analysis revealed that *sypF* and *sypG* had the highest TPM values in the uninduced wild-type sample (Fig. S1). These results argue that *sypF* and *sypG* have higher basal transcriptional levels in non-biofilm-induced conditions and suggest that they may be regulated in a distinct manner from the other genes across the locus.

Beyond the *syp* locus, *V. fischeri* encodes two additional exopolysaccharide loci (26, 40). The products of the *bcs* locus produce cellulose, which permits adhesion to surfaces and may regulate the transcription of the *syp* locus (26, 41). In both biofilm models, we observed moderate upregulation of *bcs* genes (Fig. S2A), with *bcsQ* (*VF_A0885*) and *bcsE* (*VF_A0886*) exhibiting the highest fold changes, fitting the *syp* pattern of the first gene in each respective operon being the most highly upregulated. The other exopolysaccharide locus spans *VF_0157-VF_0180* and is regulated by the quorum sensing regulator LitR (40). To date, the function of this locus remains unclear, yet it does appear to enhance phage infection by the *V. fischeri* phage HNL01 (6). In contrast to the upregulation of the *bcs* and *syp* loci within the core biofilm regulon, we noted that a majority of genes in the *VF_0157-VF_0180* EPS locus were downregulated modestly in both models (Fig. S2B).

#### Effects on other biofilm-related loci of note

**Fig 3:**
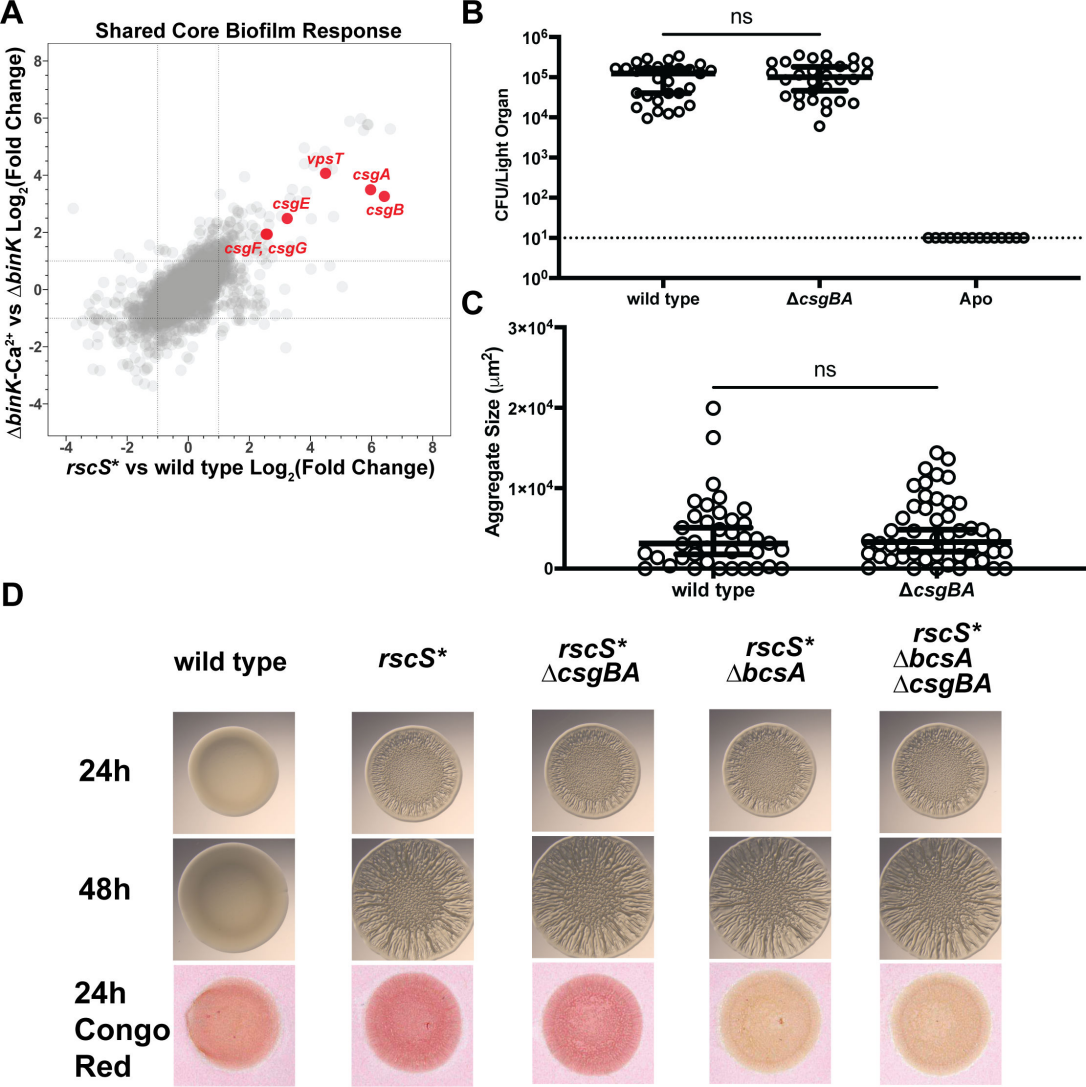
A curli locus is highly induced by both biofilm models. (A) Overlay graph of Fig. 2C with specific curli genes (red), labeled by name. (B) Single-strain colonization data for wild type and a Δ*csgBA* curli mutant. Wild type and Δ*csgBA* were inoculated into filter sterilized Instant Ocean of two separate cohorts of squid hatchlings and allowed to colonize for 3 hours. Hatchlings were then washed and allowed to grow for 2 days, after which squid juveniles were euthanized. Each dot represents the average CFU per light organ of individual squid, and horizontal bars represent median CFU per light organ with 95% confidence intervals. The dashed line indicates the limit of detection. Statistical significance was calculated using a Mann-Whitney test. (C) Biofilm aggregate size during host colonization. Wild type and Δ*csgBA* strains carrying the constitutive GFP plasmid pVSV102 were allowed to colonize squid hatchlings for 3 hours, at which point each squid was euthanized and fixed in 4% paraformaldehyde. Dissection of each squid then revealed the biofilm aggregates forming on the light organ surface, which were imaged with a Zeiss Axio Zoom fluorescence microscope. Each point represents the area of one aggregate. Squid which did not have any aggregates have points at 0. Horizontal bars represent median aggregate area size with 95% confidence interval. Statistical significance was calculated using a Mann-Whitney test. (D) Wrinkled colony and Congo Red binding assays. Strains were spotted on LBS media and imaged. ns, not significant.

Among the most highly upregulated genes in the core biofilm regulon, we noted a predicted curli amyloid fiber biosynthetic locus (Fig. 3A). In other bacteria, CsgA subunits polymerize into curli amyloid fibers on the exterior of the cell in a process nucleated by CsgB (42, 43). Curli fibers mediate aggregation and biofilm formation in different species, and the operon structure in *V. fischeri* mimics that of other organisms including predicted export genes (43–45). The *V. fischeri* CsgA and CsgB proteins are larger than their *Escherichia coli* homologs, and each shares 32.0% similarity to the characterized curli proteins from *E. coli* K-12 (46, 47). Notably, *V. fischeri* curli proteins are larger than their *E. coli* homologs, with CsgA and CsgB predicted to be 315 and 190 amino acids (2.1× and 1.25× in length, respectively). We generated a strain lacking the *csgBA* genes (Δ*csgBA*), eliminating both the major and minor curlin subunits, and queried whether interruption of the curli genes reduced host colonization levels. We observed no significant difference in final colonization levels between wild type and the Δ*csgBA* mutant (Fig. 3B), suggesting that the curli locus is not a critical colonization factor in this context. We further tested whether the absence of curli would impact biofilm aggregate formation in the host. However, we did not observe any significant reductions in the size of biofilm aggregates in the Δ*csgBA* mutant compared to wild type, suggesting that the curli locus is not required in host biofilm aggregates (Fig. 3C). Outside of the squid host, we tested whether the curli amyloid fiber locus impacted *in vitro* models of biofilm formation by introducing the Δ*csgBA* mutation into a RscS overexpression background (rscS*). We noted no significant defects in wrinkled colony formation in the rscS* Δ*csgBA* mutant at both early (24 hours) and late (48 hours) timepoints (Fig. 3D). Additionally, the Δ*csgBA* mutant did not have decreased binding of Congo Red dye, in contrast to what is observed for *csgBA* deletions in other organisms (48). Given the linkage between curli production and cellulose EPS in other organisms (49, 50), we proceeded to test whether deletion of both *csgBA* and the cellulose EPS synthase *bcsA* would impact biofilm formation. In the wrinkled colony biofilm model, the strain lacking both curli and *bcsA* demonstrated no significant differences in morphology compared to a parental biofilm-induced strain, and it exhibited Congo Red binding equivalent to a *bcsA* single mutant. Therefore, while it is intriguing that the curli system is induced concomitant with biofilm formation, we did not detect a functional requirement for the curli genes. Given our results and the lack of Congo Red binding attributable to the curli gene products, we are unable to determine whether curli fibers are produced or play a role during symbiotic colonization.

#### Effects on flagellar motility

**Fig 4:**
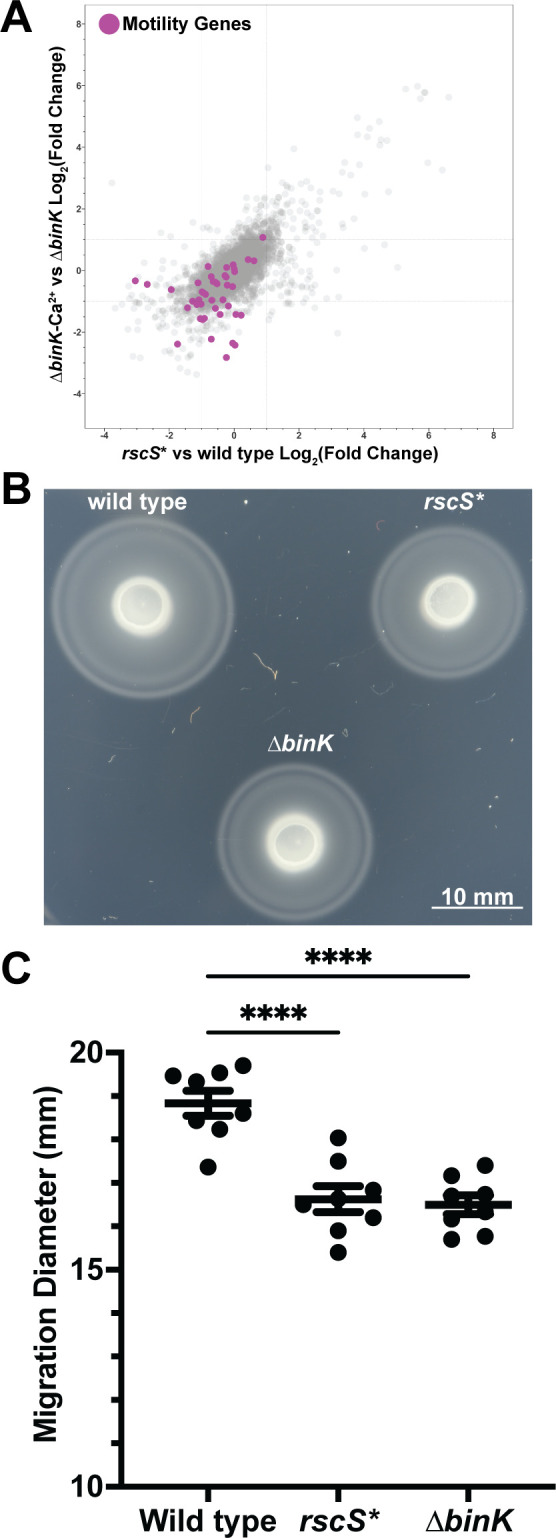
Biofilm-induced conditions downregulate flagellar motility genes and have reduced motility. (A) Both biofilm-induced models significantly downregulate genes required for flagellar biosynthesis from Brennan et al. (51). (B) Strains were cultured in TBS media, spotted onto TBS soft-agar plates, and incubated at 25°C for 5 hours. Shown is a representative plate of *n* = 8 biological replicates, imaged with a Nikon D810 camera. (C) Migration distance determined using ImageJ. Statistical significance was calculated using an ordinary one-way analysis of variance using Dunnett’s multiple comparisons test in GraphPad Prism. Each dot is the product of *n* = 3 technical replicates. Horizontal lines represent the mean with standard error of the mean. ****P < 0.0001.

Outside of exopolysaccharide regulation, the core biofilm regulon had a substantial downregulation of motility genes. We observed that 32 of the 43 genes required for swimming motility in *V. fischeri* (51) trended toward reduced expression in both biofilm models. Of these genes, five met our significance threshold: *cheB/VF_1830*, *cheY/VF_1833*, *fliN/VF_1844*, *fliM/VF_1845*, and *flrB/VF_1855* (Fig. 4A; Table S1). We therefore asked whether the rscS* or Δ*binK* backgrounds had a deficit in swimming motility by examining migration in tryptone broth salt (TBS) soft agar swim plates (Fig. 4B). Compared to wild-type controls, either rscS* or the Δ*binK* genetic backgrounds alone were sufficient to reduce swimming migration on soft agar by at least 15%, demonstrating that upregulation of the biofilm pathway leads to a diminution of swimming motility (Fig. 4C).

#### Effects on cyclic di-GMP

In *V. fischeri* and in many bacteria, increased cyclic di-GMP (c-di-GMP) levels positively impact the production of the cellulose exopolysaccharide and negatively regulate swimming motility, both effects noted in our study (41, 52). Within the transcriptomics data set, we asked which diguanylate cyclases (DGCs) and phosphodiesterases (PDEs) were differentially regulated under these conditions (Fig. S3A; Table S2). Both biofilm models (rscS* and Δ*binK*-Ca^2+^) upregulated two DGCs (*VF_A0152* and *casA/VF_1639*), two PDEs (*VF_A0526* and *binA*/*VF_A1038*), and one predicted bifunctional DGC/PDE (*VF_0985*). In contrast, both models downregulated four DGCs (*VF_1350*, *VF_A0398*, *mifB*/*VF_A0959*, and *VF_A1012*), one PDE (*VF_A0551*) and two DGCs/PDEs (*VF_0094* and *VF_A0244*). We next directly tested production of c-di-GMP using a plasmid-based fluorescent reporter (pFY4535) (53). This reporter has constitutive expression of the AmCyan fluorescent protein, with the expression of TurboRFP under the control of c-di-GMP-binding riboswitches. Higher levels of c-di-GMP therefore increase the TurboRFP:AmCyan ratio. Compared to wild-type *V. fischeri* and control strains known for either high c-di-GMP levels (Δ6PDE) or low c-di-GMP levels (Δ7DGC) (41, 52), we noted that rscS* induced a mild but significant increase in c-di-GMP levels in colony biofilms grown on standard Luria-Bertani salt (LBS) media, while no such induction was observed in the Δ*binK* background (Fig. S3B). Calcium supplementation (10 mM) of LBS media greatly increased c-di-GMP levels in both rscS* and the Δ*binK* colony biofilms to equivalent levels. This result may be explained by the shared upregulation of the calcium-induced DGC *casA* in both models, as overexpression of CasA has previously been linked to increased c-di-GMP synthesis in calcium-supplemented liquid cultures (Fig. S3C) (54). Overall, both models of biofilm formation appear to be capable of increasing the global levels of c-di-GMP.

### RscS overexpression and calcium supplementation induce regulatory programs unique to each model

Despite the contributions of both biofilm models to the shared biofilm regulon, we also noted targets that appeared to be regulated by either model alone. For the RscS overexpression model, we detected 61 genes with increased expression and 68 genes with reduced expression specific to this model (Table S3). Notable targets within the upregulated group included genes encoding the regulator HbtR/VF_A0473 and its chaperone HbtC/VF_A0474, alongside 15 genes encoded on the natural plasmid of *V. fischeri* ES114, pES100. Of the 68 downregulated genes, we observed multiple genes associated with flagellar motility, including *fliA/VF_1834*, flagellar motor protein genes *motA2/VF_A0186* and *motB2/VF_A0187*, and the chemotaxis factors *cheV2/VF_A0802* and *VF_2042*. Compared to the RscS overexpression model, the Δ*binK*-Ca^2+^ model affected fewer targets, with 38 upregulated genes and 53 downregulated genes specific to this model (Table S3). The upregulated group contained multiple iron and copper assimilation loci and the quorum sensing autoinducer C8-HSL synthase AinS. Downregulated genes in this model included multiple flagellar genes including *flaA/VF_1866*, as well as predicted regulatory factors including the cold-shock DNA-binding transcriptional regulator *cspG/VF_A1094*.

### Novel members of the core biofilm regulon are induced independently of the response regulator SypG

**Fig 5:**
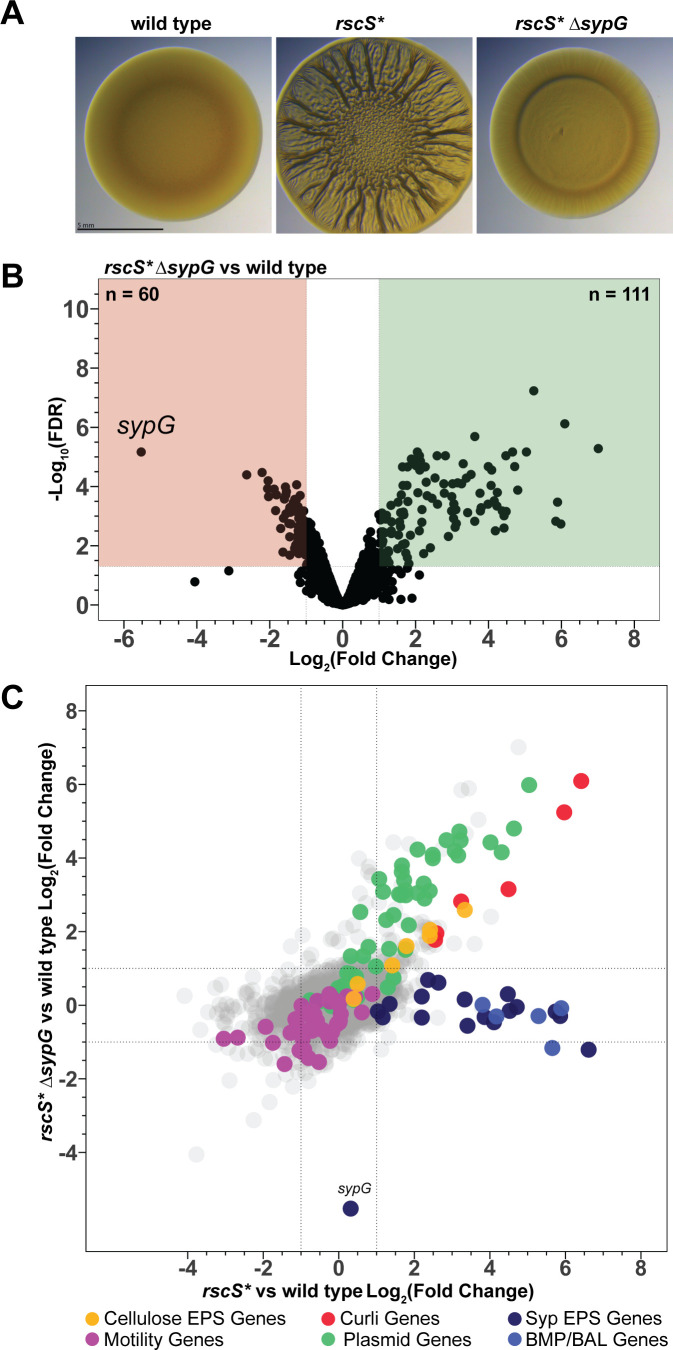
Deletion of the response regulator gene *sypG* reveals a signaling bifurcation downstream of RscS. (A) Wrinkled colony assay. (B) Volcano plot of wild type vs rscS* Δ*sypG* differential expression analysis. Green and red boxes demarcate genes with log_2_(fold change) ≥1 and ≤−1, respectively, with significant differential expression (FDR < 0.05). The numbers of genes in each box are listed in the figure. (C) Overlay of log_2_(fold change) values for all genes in both the rscS* vs wild type and the rscS* Δ*sypG* vs wild type models.

The dominant mode of signaling from sensor kinase RscS is through hybrid sensor kinase SypF to response regulator SypG (27). We therefore expected that removal of SypG in the rscS* overexpression background would eliminate most of the effect observed in that model, based on previous publications and our own colony biofilm assays (Fig. 5A) (25, 27, 55). While the *syp*, *bmp*, and *bal* genes did not exhibit induction in the absence of SypG, many of the remaining rscS*-induced genes continued to be differentially expressed without the downstream regulator (Fig. 5B). A total of 111 genes were significantly upregulated and 60 genes were significantly downregulated, despite the attenuation of the rscS* Δ*sypG* mutant in forming wrinkled colony biofilms. These results suggest that RscS maintained a regulatory program independent of SypG signaling (Fig. 5B). Visualization of these data revealed a clear bifurcation showing this unexpected SypG-independent effect within the rscS* model (Fig. 5C; Table S4).

### The SypG-independent signaling pathway regulates *bcs* locus transcription through the transcriptional regulator VpsR independent of SypF

**Fig 6:**
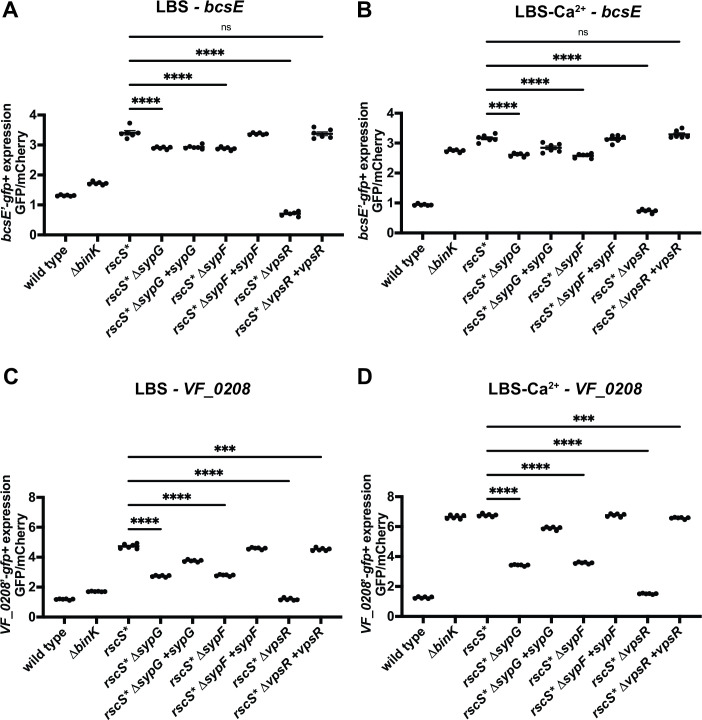
RscS uses VpsR to regulate SypG-independent targets. (A) Expression data for the *bcsE*’-*gfp*^+^ reporter for strains spotted on LBS media. Complementation backgrounds are indicated by the presence of +*vpsR*, +*sypF*, and +*sypG*; all complementations were single-copy insertions at the *V. fischeri* Tn7 transposon site. Fluorescence was measured within colony spots with *n* = 6 biological replicates, each the product of *n* = 2 technical replicates using a Zeiss Axio Zoom fluorescence microscope. GFP fluorescence was normalized to mCherry fluorescence to determine the expression of the reporter in each strain. Statistical analysis of each strain’s reporter expression was conducted in GraphPad Prism, using an ordinary one-way analysis of variance, with Dunnett’s multiple comparison test. **P ≤ 0.001, ****P < 0.0001. (B) Expression data for the *bcsE*’-*gfp*^+^ reporter for strains spotted on LBS-Ca^2+^ media, collected and analyzed as in panel A. (C) Expression data for the *VF_0208*’-*gfp*^+^ reporter for strains spotted on LBS media, collected and analyzed as in panel A. (D) Expression data for the *VF_0208*’-*gfp*^+^ reporter for strains spotted on LBS-Ca^2+^ media, collected and analyzed as in panel A. ns, not significant.

The detection of SypG-independent RscS regulation suggested that RscS was not limited to being simply a regulator of the *syp* locus. However, the factors involved in transmitting RscS signaling to alternative pathways remained unclear. One possibility was that RscS might branch a new signaling pathway through its partner sensor kinase SypF. SypF has precedent as a sensor kinase with multiple downstream response regulators, affecting both SypE and SypG within the Syp phosphorelay (27, 29). Additionally, an increased activity allele of SypF (SypF1) was discovered to increase production of the cellulose exopolysaccharide (26, 54). This activity required the gene *VF_0454*, which encodes a homolog of the *Vibrio cholerae* transcriptional regulator VpsR (26, 54). As we observed robust induction of the cellulose exopolysaccharide (*bcs*) locus in the rscS* Δ*sypG* background (vs wild type) and in the core biofilm regulon, we hypothesized that SypF-mediated VpsR signaling could explain the SypG-independent signaling pathway. To test this hypothesis, we asked whether transcription of SypG-independent gene *bcsE* was SypF and VpsR dependent. Using fluorescence microscopy, we measured reporter expression in wrinkled colony biofilms grown on LBS or LBS-Ca^2+^ agar. While removal of SypF or SypG exhibited mild effects (up to 18%), only removal of VpsR eliminated the induction of the *bcsE’-gfp^+^* reporter (Fig. 6A and B). Testing another SypG-independent promoter, for *VF_0208*, revealed roles for SypF, SypG, and VpsR, yet again VpsR exhibited the strongest impact on the promoter’s activity (Fig. 6C and D). Together, these data suggest that VpsR plays an important role in the regulation of genes in the rscS* model.

## DISCUSSION

In this work, we profiled two separate models of biofilm formation in the model symbiont *V. fischeri* ES114. This analysis revealed remarkable similarities between transcriptional responses to the two models, which facilitated identification of a core transcriptional program. In addition to known factors that were among the highest expressed in both models (e.g., *syp* locus genes), we identified novel regulatory targets that included well-annotated genes (e.g., motility), loosely annotated genes (e.g., *csg* and *VF_0157-VF_0180* EPS), as well as many hypothetical and unannotated genes. This work generated testable hypotheses, and we pursued multiple examples of those within the study. We validated that in both models, biofilm formation resulted in diminished swimming motility, consistent with behavior in other organisms in which biofilm and motility are discordantly regulated (56, 57).

Our study provided insights into the quantitative nature of induction dynamics across biofilm operons. We observed the *syp* locus had dramatically higher induction for genes located earlier in each of the four operons, as seen for *sypA*, *sypI*, *sypM*, and *sypP* (Fig. 2D). This result suggests that *sypA* provides especially high dynamic range to use for reporting on core biofilm responses in strain ES114, consistent with previous studies that have applied *sypA′-gfp*^+^ and *sypA′-lacZ*^+^ transcriptional reporters (27, 31, 34, 35, 41). In uninduced conditions, we found that the genes encoding the central regulators of the Syp phosphorelay, *sypF* and *sypG*, had the highest abundance as measured by TPM among the *syp* gene transcripts. This result suggested to us that these transcripts may be regulated in a fashion uncoupled from the preceding genes. We speculate that there is a separate regulatory mechanism that enables a higher baseline of *sypF* and *sypG* transcripts without full *syp* locus induction, and that allows levels of SypF and SypG to accumulate to be able to respond to physiological induction of the pathway. A separate, SypG-independent promoter upstream of *sypF* would be consistent with the pattern that we observed, and such a promoter was in fact proposed in early models of the *syp* locus (22, 26). In a recent study, induction of *sypF-H* in response to the biofilm stimulatory vitamin para-aminobenzoic acid was observed in a pattern distinct from the rest of the locus (32), supporting a separate regulatory mechanism that begins at *sypF*.

Among the most highly expressed genes in our biofilm models, we noted the presence of a predicted curli amyloid fiber biosynthetic locus. In other organisms, curli fibers play critical roles in biofilm formation, aiding in cell-surface attachment and cell-cell aggregation (45, 58, 59). However, we noted that this locus was dispensable during host colonization and had no observable effect in culture biofilm models or *in vivo* biofilm aggregates. Previous literature has indicated that curli functionality is commonly aided by the presence of cellulose exopolysaccharide production, which is typically regulated in tandem with curli (50, 58, 60). In our transcriptional data, we noted that the *V. fischeri* cellulose (*bcs*) and curli loci appeared to be coregulated and therefore generated a double mutant in both pathways, in the case that cellulose and curli had redundant roles in biofilm formation. However, this double mutant failed to impact wrinkled colony formation, suggesting that the functional role of curli may not have been captured in our experiments. Considering that curli do not appear to be required for host colonization, it is possible that curli may be important for *V. fischeri* survival in the marine environment in its free-living state. One possible benefit of curli production in this context may arise from the ability of curli fibers to protect producing cells from bacteriophage predation, especially within biofilms (61). Given the abundance of bacteriophage in the marine environment (62), curli production may therefore prove to be vital in the natural context but dispensable in the laboratory environment. Outside of phage defense, the basic nature of curli as adhesins may also aid *V. fischeri* in adhering to biotic and abiotic surfaces in the water column, a common behavior observed in many *Vibrio* species (63–65). Further study is needed to identify the importance of curli to *V. fischeri* biology, especially in the context of biofilm formation.

The large number of genes induced in the rscS* Δ*sypG* strain was surprising, given that the strain does not induce biofilm formation in culture. This result suggested to us that there is a substantial novel output to the RscS signaling pathway, and in the two promoters we examined, we found that VpsR was required for activity in both cases. Mutants in *vpsR* exhibit a competitive defect in squid colonization and a defect in cellulose regulation in culture (26, 66). Despite characterized VpsR regulation via SypF, in the rscS* model, we observed a substantial role for VpsR even when there was only a modest role for SypF (Fig. 6A and B). We note that additional work on VpsR has been conducted in *V. cholerae*, which has a protein that is 66% identical to the *V. fischeri* ortholog. There, it has been shown to be regulated by phosphate and not by phosphorylation as would be expected of a putative response regulator (67). Furthermore, in *V. cholerae*, VpsR regulates the *Vibrio* polysaccharide locus (*vps*), which is absent in *V. fischeri*, as similarly the *bcs* locus is absent in *V. cholerae* (26). *V. cholerae* VpsR binding sites have been determined (68), but we were unable to locate similar sites upstream of genes in our data set, suggesting that *V. fischeri* VpsR likely binds to a different consensus sequence. We also note that in *Vibrio vulnificus*, the VpsR ortholog BrpR regulates the *brp* EPS gene cluster in concert with BrpT, providing additional diversity to VpsR regulation across the *Vibrionaceae* (69). Overall, therefore, the role of VpsR in *V. fischeri* specifically and vibrios broadly requires further clarification.

Overall, this work combines two models to identify novel aspects of biofilm regulation, reveal patterns of gene expression across regulated loci, and uncover new factors that are coregulated with the *V. fischeri* symbiotic biofilm program. Our results can help to guide selection of an appropriate model given a question under consideration. The overall similarity observed suggests that most effects that have been described in the heavily studied rscS* model will carry over to the ∆*binK*-Ca^2+^ model, which is helpful, given that some phylogenetic groups of *V. fischeri* do not encode RscS (if researchers do not wish to overexpress a heterologous protein in those strains) (23, 24). Given that ∆*binK*-Ca^2+^ model substantially induced c-di-GMP levels, whereas the rscS* model does not, this is another consideration that may guide choice of model. Finally, we noted specific regulatory groups for which the two models differed from the core biofilm response. Future work can leverage these baseline data on the core response and on model-specific effects to examine temporal regulation of biofilm formation, biofilm dispersal, and regulation of biofilm behaviors during host colonization.

## MATERIALS AND METHODS

### Bacterial strains, plasmids, and media

*V. fischeri* and *E. coli* strains studied in this work are listed in Table 1. Plasmids generated or used in this work can be found in Table 2. Unless otherwise specified, *V. fischeri* strains were grown at 25°C in LBS medium (per liter: 25 g of Difco Miller LB broth [BD], 10 g of NaCl, 50 mL of 1 M Tris buffer [pH 7.5], and 10 mM CaCl_2_ [when noted]) or TBS medium (per liter: 10 g of Gibco Bacto tryptone, 20 g of NaCl, 50 mL of 1 M Tris buffer (pH 7.5), and 3 g agar). *E. coli* strains used for cloning and conjugation were grown at 37°C in Luria-Bertani medium (LB; per liter: 25 g of Difco Miller LB broth [BD]) or brain heart infusion medium (BHI; per liter: 37 g brain heart infusion powder [BD]). When needed, antibiotics were supplemented at the following concentrations: kanamycin (Kan), 100 µg/mL for *V. fischeri* and 50 µg/mL for *E. coli*; chloramphenicol (Cam), 5 µg/mL for *V. fischeri* and 25 µg/mL for *E. coli*; erythromycin (Erm), 5 µg/mL for *V. fischeri* and 150 μg/mL for *E. coli*; gentamicin (Gent), 2.5 µg/mL for *V. fischeri* and 5 µg/mL for *E. coli*. When needed for specific strains, thymidine was added at 0.3 mM for *E. coli*. Solidified media were prepared with an agar concentration of 1.5% unless specified otherwise. For Congo Red agar, 40 µg/mL Congo Red and 15 µg/mL Coomassie blue were added to LBS. Plasmids were conjugated from *E. coli* strains into *V. fischeri* using standard techniques (70).

**TABLE 1:** Bacterial strains used in this study

Strain	Genotype	Source or reference
*Vibrio fischeri*		
KV9599 = ∆7DGC	∆*VF_1200*::FRT ∆*mifA*::FRT ∆*VF_1245*::FRT ∆(*VF_A0342-VF_A0343*)::FRT ∆*VF_1639*::FRT ∆*mifB*::FRT-Spec	41
KV9601 = ∆6PDE	∆*pdeV*::FRT ∆*VF_2480*::FRT ∆*binA*::FRT ∆*VF_0087*::FRT ∆*VF_1603*::FRT ∆*VF_A0506*::FRT-Trim	41
MJM1100 = ES114	Natural isolate, *Euprymna scolopes* bobtail squid light organ	71, 72
MJM1107	MJM1100 pVSV102	73
MJM1198	MJM1100 (IG (*glpR-rscS*)::Tn*5* (Cam^R^) = rscS*)	36
MJM1538	MJM1100/pLostfoX	73
MJM2090	MJM1100/pLostfoX-Kan	73
MJM2251	MJM1100 ∆*binK*	30
MJM2479	MJM1198 *att*Tn*7*::*erm*	30
MJM3259	MJM1100 rscS* ∆*sypG*	This work
MJM3381	MJM1100 *att*Tn*7*::*erm*	This work
MJM3954	MJM1100 ∆*sypF*::*erm-bar*	This work
MJM3972	MJM1100 ∆s*ypF*::*bar*	This work
MJM4009	MJM1100/pFY4535	41
MJM4135	KV9599/pFY4535	41
MJM4137	KV9601/pFY4535	41
MJM4174	MJM1100 ∆(*VF_2409-VF_2410*)::*erm-bar*	This work
MJM4185	MJM1100 ∆(*VF_2409-VF_2410*)::*bar*	This work
MJM4269	MJM2251/pFY4535	This work
MJM4271	MJM1198/pFY4535	This work
MJM4276	MJM4185 pVSV102	This work
MJM4457	MJM1100 ∆*bcsA*::*erm-bar*	This work
MJM4467	MJM1100 ∆(*VF_2409-VF_2410*)::*bar*/pLostfoX-Kan	This work
MJM4481	MJM1100 rscS* ∆(*VF_2409-VF_2410*)::*bar*	This work
MJM4539	MJM2251 *att*Tn*7*::*erm*	This work
MJM4549	MJM1100 ∆*bcsA*::*bar*	This work
MJM4671	MJM1100 ∆*vpsR*::*erm-bar*	This work
MJM4683	MJM1100 ∆*vpsR*::*bar*	This work
MJM4915	MJM1100 ∆(*VF_2409-VF_2410*)::*bar* ∆*bcsA*	This work
MJM4917	MJM3972 rscS*	This work
MJM4918	MJM1100 rscS* ∆*bcsA*::*bar*	This work
MJM4919	MJM1100 rscS* ∆*vpsR*::*bar*	This work
MJM4956	MJM1100 rscS* ∆(*VF_2409-VF_2410*)::*bar* ∆*bcsA*	This work
MJM5034	MJM4919 *att*Tn*7*::*erm*	This work
MJM5035	MJM4919 *att*Tn*7*::*vpsR-erm*	This work
MJM5303	MJM3259 *att*Tn*7*::P*_nrdR_-sypG-erm*	This work
MJM5304	MJM4917 *att*Tn*7*::P*_nrdR_-sypF-erm*	This work
MJM5305	MJM3259 *att*Tn*7*::*erm*	This work
MJM5306	MJM4917 *att*Tn*7*::*erm*	This work
MJM5319	MJM2479/pJVG11	This work
MJM5320	MJM3381/pJVG11	This work
MJM5321	MJM4539/pJVG11	This work
MJM5322	MJM5034/pJVG11	This work
MJM5323	MJM5035/pJVG11	This work
MJM5324	MJM5303/pJVG11	This work
MJM5325	MJM5304/pJVG11	This work
MJM5326	MJM5305/pJVG11	This work
MJM5327	MJM5306/pJVG11	This work
MJM5332	MJM2479/pJVG25	This work
MJM5333	MJM3381/pJVG25	This work
MJM5334	MJM4539/pJVG25	This work
MJM5335	MJM5034/pJVG25	This work
MJM5336	MJM5035/pJVG25	This work
MJM5337	MJM5303/pJVG25	This work
MJM5338	MJM5304/pJVG25	This work
MJM5339	MJM5305/pJVG25	This work
MJM5340	MJM5306/pJVG25	This work
*Escherichia coli*		
MJM637	S17-1 λpir/pUX-BF13	74
MJM537	DH5α λpir	Laboratory stock
MJM3999	NEB5α/pFY4535	53
MJM2090	MJM537/pLostfoX-Kan	73
MJM658	BW23474/pEVS107	75
MJM4881	MJM537/pKK01	This work
MJM4879	MJM537/pJVG21	This work
MJM5026	MJM537/pJVG23	This work
MJM5025	MJM537/pJVG22	This work
MJM4640	MJM537/pJVG11	This work
MJM5235	MJM537/pJVG25	This work
MJM3478	π3813/pKV496	76
MJM542	MJM537/pVSV102	77
MJM534	CC118 λpir/pEVS104	70
MJM570	DH5α/pEVS79	70
MJM687	MJM537/pMarVF1	73
MJM3271	NEB5α/pEVS79-Erm	This work
MJM4890	MJM537/pKMB036	This work
MJM689	S17-1 λpir/pTM267	78

**TABLE 2:** Plasmid list

Plasmid	Description	Source or reference
pUX-BF13	Tn7 transposase helper plasmid (*tns* genes)	74
pFY4535	Cyclic di-GMP reporter plasmid, Gent^R^	53
pLostfoX	TfoX-plasmid for enabling efficient transformation, Cam^R^	79
pLostfoX-Kan	TfoX-plasmid for enabling efficient transformation, Kan^R^	73
pEVS107	Mini-Tn*7* transposon backbone vector, Kan^R^ Erm^R^	75
pKK01	pEVS107 *vpsR* (±300bp), Kan^R^ Erm^R^	This work
pJVG21	pEVS79-Erm rscS* (±1600 bp from the MJM1198 background), Erm^R^ Cam^R^	This work
pJVG22	pKMB036 P*_nrdR_*-*sypF*	This work
pJVG23	pKMB036 P*_nrdR_*-*sypG*	This work
pTM267	Promoterless *gfp* with constituitive-expressed mCherry for transcriptional reporters	78
pJVG11	pTM267 *bcsE'-gfp^+^*	This work
pJVG25	pTM267 *VF_0208'-gfp^+^*	This work
pKV496	pEVS79 encoding FLP recombinase, Kan^R^	76
pVSV102	Consitutive GFP for squid colonization assays, Kan^R^	77
pEVS104	Helper plasmid for conjugations, Kan^R^	70
pEVS79-Erm	Vector for allelic exchange, Erm^R^	This work
pEVS79	Vector for allelic exchange, Cam^R^	70
pMarVF1	*V. fischeri* Mariner transposon vector, Amp^R^ Erm^R^	73

### DNA synthesis and sequencing

Primers used in this work are provided in Table 3 and were synthesized by Integrated DNA Technologies (Coralville, IA). PCR products of gene deletions and plasmid modifications were validated using Sanger sequencing at Functional Biosciences (Madison, WI). Sequencing results were analyzed using Benchling. All Sanger sequencing products were generated with Q5 High-Fidelity polymerase (NEB), Phusion Hot Start Flex DNA polymerase (NEB), or OneTaq DNA polymerase (NEB). Diagnostic PCRs were performed with OneTaq DNA polymerase (NEB) or GoTaq DNA polymerase (Promega). Whole-plasmid sequencing was performed by Plasmidsaurus (Eugene, OR) when noted.

**TABLE 3:** Primer list

Primer	Sequence (5′−3′)	Notes
DAT_015	accaagaagcagtacgacgattat	Internal primer in *rscS* directed toward the promoter region
DAT_016	gaatcaactcaagaaactccccta	Primer external to *rscS* promoter region, used to confirm insertion of rscS* with DAT_015
DAT_017	ggcagaatgcttaatgaattacaa	Primer in rscS* allele chloramphenicol cassette directed toward the start of the gene, verifies presence of rscS* allele with DAT_015
DAT_018	taaataaatcctggtgtccctgtt	Primer to confirm insertion of pLostfoX-Kan, paired with DAT_018
DAT_019	tcgctgttaaaaggacaattacaa	Primer to confirm insertion of pLostfoX-Kan, paired with DAT_019
HB42	acgagacgagcttcttatatatgcttcgccag	Generates erythromycin cassette for ∆*gene*::*erm-bar* backgrounds.
HB154	cgatcttgtgggtagagacatccaggtcaagtcnnbnnbnnbnnbnnbnnbggaatcaagtgcatgagcgctgaag	Generates erythromycin cassette for ∆*gene*::*erm-bar* backgrounds, generates random barcode for strain labeling. Paired with HB142
JVG_AH1	tccagataatgccgtcggtc	Primer 1 kb upstream of *csgB*, used to generate flanking region for *csgBA*::*erm-bar*
JVG_AH2	agcatatataagaagctcgtctcgtcatatcagtatccctctttcgtgatagc	Primer paired with JVG_AH1 to generate *csgB* upstream flanking region for *csgBA*::*erm-bar*, has homology to HB42 and HB154 amplified erythromycin cassette
JVG_AH4	gctgagcaagcgactgataac	Primer located 1 kb downstream of *csgB*, used to verify *csgBA*::*bar* flippase treatment with JVG_AH1
JVG_AJ0	ctcgctaattattttacgactaagcc	Primer located ~650 bp upstream of *csgB*, used to validate *csgBA*::*erm-bar* with HB08
JVG_AJ1	aataatcgctcgaccagagg	Primer located 1 kb upstream of *csgA*, used with JVG_AJ4 to validate deletion of *csgBA*::*erm-bar*
JVG_AJ3	ggatgtctctacccacaagatcggttgtaattactcaactataaatttatcacttaaat	Primer paired with JVG_AJ4 to generate *csgA* downstream flanking region for *csgBA*::*erm-bar*. Has homology to HB42 and HB154 amplified erythromycin cassette
JVG_AJ4	gccagctcaatacttcctacc	Primer 1 kb downstream of *csgA*, used to generate flanking region for *csgBA*::*erm-bar*
JVG_AJ5	cagctgatgtagagctatttaatgcag	Primer internal to *csgA*, used to confirm *csgBA*::erm-bar
JVG_AJ6	ccattttccaatgtaacctcagcc	Primer internal to *csgA*, used to confirm *csgBA*::*erm-bar*, paired with AJ5
RYI554	gccagttggtgttatcactatggttt	Primer internal to *bcsA*, used to confirm *bcsA*::*erm-bar*, paired with RYI555
RYI555	tcgaaacatcataccaagagaccataatgc	Primer internal to *bcsA*, used to confirm *bcsA*::*erm-bar*, paired with RYI554
RYI556	ggattcaatatccagtaaggaataggttatctct	Primer 1 kb upstream of *bcsA*, used to generate flanking region for *bcsA*::*erm-bar*.
RYI557	agcatatataagaagctcgtctcgtcatagattatttaccctctacctctttcgcc	Primer paired with RYI556 to generate *bcsA* upstream flanking region for *bcsA*::*erm-bar*, has homology to HB42 and HB154 amplified erythromycin cassette
RYI558	ggatgtctctacccacaagatcgccaaaaaaagtcaatttttagagtgcaatcagt	Primer paired with RYI559 to generate *bcsA* downstream flanking region for *bcsA*::*erm-bar*, has homology to HB42 and HB154 amplified erythromycin cassette
RYI559	tgccgatggtcctgtaagtagatc	Primer 1 kb downstream of *bcsA*, used to generate downstream flanking region for *bcsA*::*erm-bar*
RYI560	gatatcaatgaggtaatgcttttattttttgcgaa	Primer outside upstream flanking region of *bcsA* for validating deletion
RYI561	gcataattcttacttggttgcatggtttg	Primer outside downstream flanking region of *bcsA* for validating deletion
JVG_BJ9	gcaagacctaaccctgtaccac	Primer for testing insertion of the rscS* promoter allele, pairs with JVG_BJ10
JVG_BJ10	tttactaagcgaatattgcgaggag	Primer for testing insertion of the rscS* promoter allele from pJVG21 into the *rscS* promoter, pairs with JVG_BJ9
KK017	tagttaataacaataagcaagttcgctggatg	Primer internal to *vpsR*, used to confirm *vpsR*::*erm-bar*, paired with KK018
KK018	ctaggtgctcaagatcaatcgtcatg	Primer internal to *vpsR*, used to confirm *vpsR*::*erm-bar*, paired with KK017
KK019	gagtatgtactgctaacaaaagcattacgtc	Primer outside upstream flanking region of *vpsR* for validating deletion
KK020	gtcagacctacctcttcgtataattcacg	Primer outside downstream flanking region of *vpsR* for validating deletion
KK021	cgacaaaatgccttatggtgagca	Primer 1 kb upstream of *vpsR*, used to generate flanking region for *vpsR*::*erm-bar*
KK022	agcatatataagaagctcgtctcgtcataacactacctctaaattcttatatctaaaccaacttaaa	Primer paired with KK021 to generate *vpsR* upstream flanking region for *vpsR*::*erm-bar*, has homology to HB42 and HB154 amplified erythromycin cassette
KK023	ggatgtctctacccacaagatcggagccgagaagctatagctaatcga	Primer paired with KK024 to generate vpsR downstream flanking region for *vpsR*::*erm-bar*. Has homology to HB42 and HB154 amplified erythromycin cassette
KK024	gctttgatcttttccattaaaaagtgattggat	Primer 1 kb downstream of *vpsR*, used to generate flanking region for *vpsR*::*erm-bar*
RYI193	cggtgagagtattgcgccaat	Upstream primer for confirming *sypF*::*erm-bar* deletions
RYI194	aacaccgttgatttaattatctcagatattcaaatgc	Primer 1 kb upstream of *sypF*, used to generate upstream flanking region for *sypF*::*erm-bar*
RYI195	gacttgacctggatgtctctacccacaagatcgaaacaaggtttctcaaaataaaagaataaaacaaaattcg	Primer paired with RYI194 to generate *sypF* upstream flanking region for *sypF*::*erm-bar*, has homology to HB42 and HB154 amplified erythromycin cassette
RYI196	ctggcgaagcatatataagaagctcgtctcgtcatagttagctcctttgcaatgtttgc	Primer paired with RYI196 to generate *sypF* downstream flanking region for *sypF*::*erm-bar*, has homology to HB42 and HB154 amplified erythromycin cassette.
RYI197	gtggtggcatatgaataggcacaa	Primer 1 kb downstream of *sypF*, used to generate downstream flanking region for *sypF*::*erm-bar*
RYI198	ggactcgtcaattttgcataatgcatc	Internal primer in *sypF* to confirm deletion
RYI199	ccgatagatgatatatcaacttgtgctcct	Internal primer in *sypF* to confirm deletion, paired with RYI199
RYI200	taagagaacggcagcattaagaacattac	Downstream primer for confirming *sypF*::*erm-bar* deletions
Tn7 Site F	tgttgatgataccattgaagctaaa	Used to amplify insertions into the *V. fischeri* Tn7 insertion site, used with Tn7 Site R
Tn7 Site R	cttgctgtatgtatttgctgatga	Used to amplify insertions into the *V. fischeri* Tn7 insertion site, used with Tn7 Site F
JVG_BK13	aagggacataggcaggcatc	Sanger sequencing primer to analyze Tn7 transposons carrying the P*nrdR* promoter
JVG_BK14	tgcagagatctactagtggcc	Sanger sequencing primer to analyze Tn7 transposons carrying the P*nrdR* promoter
JVG_AA5	gggtaatgactctctagcttgagg	Primer to validate insertions into pTM267 plasmid at the MCS proximal to *gfp*, used with JVG_AA6
JVG_AA6	cgtatgtagcatcaccttcacc	Primer to validate insertions into pTM267 plasmid at the MCS proximal to *gfp*, used with JVG_AA5
RYI446	tcgaggtacctggccactagtagatctctg	Primer to linearize pEVS107 to allow use in gibson assembly with various inserts, used with RYI446
RYI447	ggcgcgcctagggccctctaga	Primer to linearize pEVS107 to allow use in gibson assembly with various inserts, used with RYI447
RYI643	tagagggccctaggcgcgcccgctctttttttatttatcttactcaaagcact	Primer to amplify *vpsR* ± 300 bp for insertion into pEVS107, used with RYI644
RYI644	ctagtggccaggtacctcgacttctttggaagaagaacgacagttaaaaca	Primer to amplify *vpsR* ± 300 bp for insertion into pEVS107, used with RYI643

### RNA isolation from *V. fischeri* wrinkled colonies

Strains MJM1100, MJM1198, MJM2251, and MJM3259 were streaked on LBS agar and grown overnight at 25°C (30, 36, 71, 72). Overnight cultures were prepared in triplicate from independent colonies of each strain in LBS media, and grown overnight at 25°C with rotation. From each overnight culture, 500 µL was spotted onto LBS agar, or LBS-Ca^2+^ agar, and spots were allowed to grow for 48 hours at 25°C. After 48 hours, each spot was collected into a 1:2 mix of LBS and RNAprotect Bacteria reagent (Qiagen), incubated at RT for 5 minutes, followed by vortexing at maximum speed on a Vortex Genie-2 (Scientific Industries), followed by centrifugation for 10 minutes at 5,000 x *g* at room temperature. Supernatants were removed, and cell pellets were stored at −80°C. RNA extraction was performed using an RNeasy PowerBiofilm kit (Qiagen), as per manufacturer instructions. Purified RNA was stored at −80°C, followed by a further on-column DNAse treatment using RNAse-free DNAse (Qiagen) followed by purification with the RNeasy MinElute kit (Qiagen). Samples were then transferred to the University of Wisconsin - Madison Biotechnology center.

### Library preparation, sequencing, and analysis of RNA samples

Samples were quality-tested using both NanoDrop and Agilent Bioanalyzer measurements. Ribosomal RNA depletion with additional probes recommended by Illumina (Table 4), stranded library preparation (Illumina Ribo-Zero Plus rRNA Depletion w/ Stranded Total RNA), and paired-end 2 × 150 sequencing was conducted at the UW-Madison Biotechnology center on an Illumina NovaSeq 6000. Table S5 shows quality control data for these samples. The resulting reads were processed by FASTP v0.20.1 (80), mapped with BWA-MEM v0.7.17 (81), read counts per gene were enumerated with HTSeq v0.13.5 (82) to *V. fischeri* ES114 chromosome 1, 2, and the natural plasmid pES100 (CP000020.2, CP000021.2, and CP000022.1), and differential expression analysis was conducted with EdgeR v3.32.1 (83). Follow-up analysis of the data set was performed in RStudio v1.3.959. Volcano plots of data were produced through plotting the −log_10_ (FDR) on the graph *y*-axis and the log_2_(fold change) on the *x*-axis, from the EdgeR results. Overlay graphs of multiple differential expression analyses were generated by plotting the log_2_(fold change) for each gene in the stated differential expression analyses on either the *x*- or *y*-axis. Transcripts per kilobase million (TPM) values for all genes were obtained from the raw read counts for all genes from HT-Seq output (39). The read counts for each gene were then divided by each gene’s respective length in kilobase pairs to generate reads per kilobase (RPK). These values were summed for each of the replicates and divided by 1,000,000 to generate the scaling factor for each replicate. The RPK for each gene was then divided by its respective replicate scaling factor to generate per gene TPM values. TPM values for all conditions can be found in Table S6.

**TABLE 4:** Supplemental rRNA depletion probes in pool vfischeri_RZP_sup_pool

Probe	Sequence
Probe 01	GCTAGAGATCGTCGCCTTGGTGAGCTCTTACCTCACCAACTAGCTAATCT
Probe 02	TAGCGCGAGAGGCCCGAAGGTCCCCCTCTTTGGTCCGAAGACATTATGCG
Probe 03	TCCAATAGTTATCCCCCACACTAAGGCATATTCCCAGGCATTACTCACCC
Probe 04	CCCTTAACGTTCCCCGAAGGTTCAGTTAAGTCGTTTCCGCTCGACTTGCA
Probe 05	ACGTCAAATGCTGCAGCTATTAACTACAACACCTTCCTCCCTACTGAAAG
Probe 06	AGGCCTTCTTCATACACGCGGCATGGCTGCATCAGGCTTTCGCCCATTGT
Probe 07	GAAATTCTACCCCCCTCTACAGCACTCTAGTTCACCAGTTTCAAATGCGG
Probe 08	AGTTTTAATCTTGCGACCGTACTCCCCAGGCGGTCTACTTAACGCGTTAG
Probe 09	AACATTTCACAACACGAGCTGACGACAGCCATGCAGCACCTGTCTCAGAG
Probe 10	TCTCCCGGTTCGCCTCGCTACGCTATGTATTCACGTAGCGATACGTGCTT
Probe 11	CCCATTCAGAAATCCCAGACTCAAATGGTTTTTACTACCTAATCTGGGCT
Probe 12	CGTCTTTCATCGCCTCTGACTGCCAAGGCATCCACCGTGTACGCTTAGTC
Probe 13	GTGTCCCGCCTTACTCGTTTTCACTGATGATGAGATGTCGGTTACGGGGC
Probe 14	CGGCACTTTCCAGAGCCTTCACCTGTCTCATTAAAAGCTTAAGGGCTAAC
Probe 15	AATCTTCAACCTGCCCATGGCTAGATCACCTGGTTTCGGGTCTATATCCA
Probe 16	GTTAAGACTCGGTTTCCCTACGGCTCCCCTAAACGGTTAACCTTGCCACT
Probe 17	ACCCATTATACAAAAGGTACGCAGTCACACCACGAAGGTGCTCCTACTGC
Probe 18	ACGACGGACGTTAGCACCCGCCGTGTGTCTCCCGGATAGTACTTACTGGT
Probe 19	AGGGTTGGTAAGTCGGGATGACCCCCTAGCCTTAACAGTGCTCTACCCCC
Probe 20	AGGCTCTACCTAAATAGATTTCGGGGAGAACCAGCTATCTCCAGGTTTGA
Probe 21	GAGCGGGTTTTTCACCCGCTTTATCGTTACTCATGTCAGCATTCGCACTT
Probe 22	CGCTTTACAACGCACCTTCAACCGCTTACAGAACGCTCCCCTACCCAATA
Probe 23	CCTGTGTCGGTTTGGGGTACGATTCCTTACAATCTGAAGCTTAGAGGCTT
Probe 24	CATCAATGGCTTCACTACCGTAGTAGCTCGACATCGTATCTCAGCCTAGT
Probe 25	CCTAAGAATACAGCCTACATACTTGAACTTGGACGACCGTCGCCAAGCCC
Probe 26	TCCCCCCATCGCAATTGTAAGAAGTACGGGAATATTAACCCGTTTCCCAT
Probe 27	TCATCTTACGATTTAGCACAGTGCTATGTTTTTAATAAACAGTTGCAGCC
Probe 28	CTCCCGGCAGCTTAGAGAGCAAGTCTCATCACCGCTAGGAGCGTACCTTC
Probe 29	GATCACTATGACCTGCTTTCGCACCTGCTCGAATTGTCATTCTCGCAGTC
Probe 30	ATTGCACTAACCTCACGATGTCCAACCGTGATTAGCCCACCTTCGTGCTC
Probe 31	GGAGGAGACCGCCCCAGTCAAACTACCCACCAGGCACTGTCCGCAATCCC
Probe 32	TTAGAACATCAAGCATACAAGGGTGGTATTTCAAGATTGCCTCCACACAT
Probe 33	TCAAAGGCTCCCACCTATCCTACACATGTAGGGTCAATGTTCAGTGCCAA
Probe 34	ATGGTTAAGCCTCACGGGCAATTAGTATCAGTTAGCTCAATGCCTCGCAG
Probe 35	CCTATCTACGTCGTAGTCTCCAACAACCCTTTAGGATACTTAAAGTATCA
Probe 36	AGGGCTCGCTTCACGCTTAGATGCTTTCAGCGTTTATCGATTCCGAACTT
Probe 37	CCACTGGCGTGACAACCCGAACACCAGAGGTTCGTCCACTCCGGTCCTCT

### Construction of pTM267-based transcriptional reporters (pJVG11 and pJVG25)

Reporter plasmids were created through insertion of gene specific promoter elements into the established transcriptional reporter plasmid pTM267 (78). In short, ~500 bp of upstream DNA preceding the start codon for *bcsE* and *VF_0208* were amplified using Q5 DNA Polymerase (NEB) using primers JVG_BC1 and JVG_BC2 (*bcsE*) and JVG_BM1 and JVG_BM2 (*VF_0208*). Each primer set appended XbaI and XmaI restriction sites to promoter amplicons. Inserts and the pTM267 backbone were then digested with XbaI and XmaI (NEB), gel purified using the QIAquick gel extraction kit (QIAgen), and further concentrated using a DNA Clean and Concentrator kit (Zymo Research). Purified inserts and pTM267 backbone were then ligated together using overnight ligation at 16°C with T4 DNA ligase (NEB). Ligation products were then transformed into chemically competent *E. coli* DH5ɑ λpir, and chloramphenicol resistant colonies were selected from plates. Potential candidates were streak purified, and used in colony PCR using primers JVG_AA5 and JVG_AA6 to amplify across the inserted element. Candidates showing the proper size amplicons were miniprepped and either sent for Sanger sequencing using primers JVG_AA5 and JVG_AA6 (pJVG11-*bcsE*) (Functional Biosciences) or for full plasmid sequencing (pJVG25-*VF_0208*) (Plasmidsaurus).

### Construction of *gene*::*bar* tagged gene deletions

The creation of Δ*gene*::*bar* mutants followed previously published protocols (84). In short, 1 kb regions flanking the gene(s) of interest were amplified from MJM1100 genomic DNA using Phusion Hot Start Flex polymerase (Thermo Fisher), and each flanking element shared terminal homology with an erythromycin resistance cassette amplified from plasmid pHB01 using primers HB42 and HB154. Splicing by overlap extension PCR (SOE-PCR) was then used to join the flanking regions onto the Erm^R^ cassette (84). This joined fragment was used as mutagenic DNA in a natural transformation into *V. fischeri* strains containing the pLostfoX or pLostfoX-Kan plasmid (73). Recombinants were selected for erythromycin resistance, streak purified, and PCR assays were used to identify candidates with insertions of the erythromycin resistance cassette in the place of targeted genes. For candidates passing PCR screens, the gene deletion and adjacent upstream and downstream regions were PCR amplified, and amplicons were sent for Sanger sequencing using primers HB08, HB09, HB42, and HB146. Candidates which successfully replaced the target gene with the erythromycin cassette were then saved as Δ*gene*::*erm-bar* strains. In order to eliminate potential influences of the erythromycin cassette on neighboring genes, the flippase-encoding plasmid pKV496 was conjugated into Δ*gene*::*erm-bar* strains via tri-parental mating with strains MJM534 and MJM3478 (76). Following this conjugation, candidates were validated for loss of the erythromycin cassette using PCR with primers flanking the deletion site, and amplicons were sent for Sanger sequencing for final validation. Candidates passing the deletion check were then saved as Δ*gene*::*bar* strains.

### Natural transformation of *V. fischeri* with genomic DNA

When necessary, plasmids pLostfoX or pLostfoX-Kan were conjugated into *V. fischeri* strains to induce competence to transfer alleles between strains (73, 79). Natural transformation was conducted as described previously (84), in which strains carrying pLostfoX or pLostfoX-Kan were grown from glycerol stocks in LBS with their respective antibiotics. After overnight growth in LBS, a 1/100 subculture was performed into Tris minimal media (TMM) containing the antibiotic of note, with further growth overnight. The following morning, the TMM overnight culture was subcultured 1/20 into fresh TMM and allowed to grow to OD600 0.2. This culture was then separated into 500 µL aliquots, and 2.4 µg genomic DNA was then provided to the aliquots as needed, and the cell/DNA mixtures were gently vortexed before resting at room temperature for 30 minutes. Following room temperature incubation, each aliquot was provided with 1 mL of LBS, and allowed to recover overnight at 25°C. Recovery cultures were then plated on LBS agar containing respective antibiotics, and candidates of note were streak purified for downstream confirmation.

### Construction of Tn7 transposon-based chromosomal complementations of *sypF*, *sypG*, and *vpsR*

Chromosomal complementations of Δ*gene*::*bar* mutations were conducted through provisioning of the gene of interest on a Tn7 transposon. For the Δ*vpsR*::*bar* strain, *vpsR* ± ~ 350 bp was amplified from MJM1100 gDNA using primers RYI643 and RYI644, and the Tn7 transposon vector pEVS107 was linearized using primers RYI446 and RYI447 with Q5 DNA polymerase (NEB) (75). Using the NEB HiFi DNA assembly kit, the *vpsR* insert and pEVS107 backbone were joined, and the products of this reaction were transformed into *E. coli* DH5ɑ λpir, and erythromycin resistant colonies were selected. Candidates were streak purified, plasmids were isolated using the QIAprep Spin Miniprep kit (Qiagen) and submitted to Plasmidsaurus for full plasmid sequencing, yielding plasmid pKK01. For the Δ*sypF* and Δ*sypG* complementations, the ORFs for each gene were amplified with JVG_BK7 and JVG_BK8 (*sypF*) and JVG_BK11 and JVG_BK12 (*sypG*) with Q5 DNA polymerase (NEB). A variant of pEVS107 containing the constitutively active promoter for the *nrdR* gene (pKMB036) was linearized using primers containing homology for *sypF* (JVG_BK5 and JVG_BK6) and *sypG* (JVG_BK9 and JVG_BK10), and the plasmid backbone and inserts were joined by the NEB HiFi DNA assembly kit. Candidates were selected as erythromycin resistant colonies, and streak purified. Successful assemblies were confirmed using primers JVG_BK13 and JVG_BK14 across the insertion site using colony PCR, plasmids were isolated using the QIAprep Spin Miniprep kit (Qiagen), and purified plasmids were sent to Plasmidsaurus for full plasmid sequencing, yielding plasmids pJVG22 and pJVG23.

### Chromosomal insertion of Tn*7* complementations of *sypF, sypG,* and *vpsR*

The Tn*7* transposon complementation plasmids of *sypF* (pJVG22), *sypG* (pJVG23), and *vpsR* (pKK01) were inserted into their respective *V. fischeri* Δ*gene*::*bar* deletion backgrounds using standard conjugation protocols, with the addition of the Tn*7* transposase helper strain MJM637, carrying plasmid pUX-BF13 (70, 74). Candidates were selected as erythromycin resistant colonies, streak purified, and PCR confirmed using primers Tn*7* site F and Tn*7* site R with Phusion HS DNA polymerase. Tn*7* site F/R amplicons were then purified using the QIAquick PCR purification kit and sent for Sanger sequencing using primers Tn*7* site F, Tn*7* site R, RYI460, and RYI461 (*vpsR*) or Tn*7* site F, Tn*7* site R, JVG_BK13, and JVG_BK14 (*sypF* and *sypG*) (Functional Biosciences). Passing candidates were then saved as MJM5035 (rscS* Δ*vpsR*::*bar att*Tn*7*::*vpsR-erm*), MJM5303 (rscS* Δ*sypG*::*bar att*Tn*7*::P*_nrdR_-sypG-erm*), and MJM5304 (rscS* Δ*sypF*::*bar att*Tn*7*::P*_nrdR_-sypF-erm*)

### Construction of rscS* allelic exchange plasmid (pJVG21)

Plasmid pEVS79 (70) modified with Erm^R^ replacing Cam^R^ (pEVS79-Erm, Matthew Hauserman) was amplified using primers JVG_BJ1 and JVG_BJ2, and the intergenic region containing the rscS* allele was amplified from strain MJM1198 using primers JVG_BJ3 and JVG_BJ4. Products of the JVG_BJ1 and JVG_BJ2 reaction were digested prior to assembly using Dpn1 (NEB). The NEBuilder HiFi DNA Assembly kit (NEB) was then used to assemble JVG_BJ1/2 and JVG_BJ3/4, products of this reaction were then transformed into *E. coli* DH5α using heat shock, and transformants were selected on BHI-Erm150 media. Candidates were screened using PCR with primers JVG_BJ5 and JVG_BJ6, passing candidates were then submitted to Plasmidsaurus (Eugene, OR) for whole-plasmid sequencing yielding plasmid pJVG21.

### Allelic exchange of rscS* into Δ*sypF,* Δ*vpsR,* Δ*bcsA,* and Δ*csgBA* Δ*bcsA*

Plasmid pJVG21 was conjugated into MJM3972 (ES114 Δ*sypF*), MJM4549 (ES114 Δ*bcsA*), MJM4683 (ES114 Δ*vpsR*), and MJM4915 (Δ*bcsA* and Δ*csgBA*) using standard laboratory protocols. Conjugation spots were scrapped from plates, resuspended in 70% IO, and plated on LBS-Erm plates to select for plasmid integration into the host genome. Candidates were streaked on LBS-Erm to purity, and individual colonies were patched on LBS-Erm, LBS-Cam, and LBS. Candidates showing Erm^R^, Cam^R^, and growth on LBS were then cultured in LBS-Erm and saved as single recombinants. Double recombinants were then generated as follows. Single recombinants were first cultured overnight in LBS; overnight cultures were then diluted by 1/100 into fresh LBS and allowed to grow until OD600 reached 0.2. At this point, a further subculture was prepared by repeating the 1/100 dilution into LBS and allowing the culture to grow once again to OD600 0.2. These subcultures were then plated on LBS media and allowed to grow overnight at 25°C. Resulting colonies were patched on LBS-Erm, LBS-Cam, and LBS and grown overnight at 25°C. Patches showing Erm^S^ dropped the plasmid backbone, yet retained the Cam^R^ of the rscS* allele were then cultured and saved as candidate double recombinants. To confirm insertion of rscS*, check PCRs were conducted using primers DAT_015 and DAT_016 and Phusion HS polymerase. Amplicons showing the proper size of the rscS* insertion were purified using the QIAgen Quickspin PCR purification kit and sent to Functional Biosciences for Sanger sequencing confirmation. Passing candidates were saved as MJM4917 (rscS* Δ*sypF*), MJM4918 (rscS* Δ*bcsA*), MJM4919 (rscS* Δ*vpsR*), and MJM4956 (rscS* Δ*bcsA* Δ*csgBA*).

### Wrinkled colony assays

Bacterial strains were grown in LBS medium for approximately 17 hours, and 8 µL of each overnight culture was then spotted onto LBS or LBS-Ca^2+^ plates, and incubated at 25°C. At 24 and 48 hours post-spotting, plates were imaged using a Leica M60 microscope and Leica DFC295 camera.

### Plasmid-based expression assay

Bacterial strains were grown in LBS-Cam medium for approximately 17 hours at 25°C, and 8 µL of each overnight culture was then spotted onto LBS-Cam and LBS-Cam-Ca^2+^ agar, followed by incubation at 25°C. After 24 hours, colony spots were imaged on a Zeiss Axio Zoom v.16 large-field 561 fluorescent stereo microscope, and fluorescence levels were measured within colony spots using the Zen Blue software. Expression of the reporter (eGFP fluorescence, 488 nm excitation, 509 nm emission, and 300 ms exposure time) was normalized to a constitutive locus on the pTM267 backbone (mCherry fluorescence, 558 nm excitation, 583 nm emission, and 700 ms exposure time) as eGFP fluorescence/mCherry fluorescence. Data analysis was conducted in GraphPad Prism using an ordinary one-way analysis of variance (ANOVA) with multiple comparisons.

### Soft agar swimming motility assay

Bacterial strains were streaked on TBS agar 48 hours prior to the assay and incubated at 25°C. After 24 hours, four single colonies were cultured into TBS medium and grown at 25°C for 17 hours with rotation. Ten microliters of each overnight culture was then spotted onto TBS motility media (3 g/L agar) in technical triplicate and allowed to incubate at 25°C. After 5 hours, plates were imaged using a Nikon D810 digital camera, and ImageJ was used to quantify the Feret diameter of each migration spot as the migration distance. Each biological replicate was produced through averaging the technical replicates. Data analysis was conducted in GraphPad Prism using an ordinary one-way ANOVA with multiple comparisons. This assay was conducted on two separate days for a total of eight biological replicates.

### Congo Red assay

Bacterial strains were grown in LBS medium for 17 hours, and 8 µL of each overnight culture was spotted on LBS Congo Red media. Plates were incubated overnight at 25°C for 24 hours, after which colony spots were transferred to printer paper, air dried, and imaged as TIFF files (76).

### Cyclic di-GMP reporter assay

Bacterial strains were grown in LBS-Gent at 25°C for 16 hours, and 8 µL of each overnight culture was spotted in technical duplicate on LBS-Gent and LBS-Gent-Ca^2+^ plates. After 24 hours, colony spots were imaged on a Zeiss Axio Zoom v.16 large-field 561 fluorescent stereo microscope, and fluorescence levels were measured within colony spots using the Zen Blue software. Expression of the reporter (TurboRFP fluorescence, 553 nm excitation, 573 nm emission, and 150 ms exposure time) was normalized to a constitutive locus on the pFY4535 backbone (AmCyan1 fluorescence, 467 nm excitation, 496 nm emission, and 150 ms exposure time) as TurboRFP fluorescence/AmCyan1 fluorescence. Data analysis was conducted in GraphPad Prism using an ordinary one-way ANOVA with multiple comparisons.

### Curli mutant squid single-strain colonization assay

Lab-reared *E. scolopes* hatchlings were colonized using a previously published protocol (85). In short, 3–5 × 10^3^ CFUs of the wild-type strain MJM1100 or the Δ*csgBA* mutant strain MJM4185 were provided to squid hatchlings, followed by a 3 hour colonization period. After 48 hours, hatchlings were measured for luminescence using a Promega GloMax 20/20 luminometer and were euthanized. Homogenization of squid tissues was carried out as in the protocol, and colonization levels were enumerated from CFU per light organ plated on LBS agar. This experiment was conducted in biological triplicate. A Mann-Whitney test was then used to determine statistical significance between the colonization levels of MJM4185 and wild type in GraphPad Prism.

### Curli mutant squid aggregation assay

Squid aggregation experiments were performed as described previously (86). In short, cultures of MJM1107 and MJM4276 were grown overnight at 25°C in LBS-Kan (77). The following morning, 10^5^–10^6^ CFU/mL of each strain was added to bowls of *E. scolopes* hatchlings and allowed to colonize for 3 hours. Following this colonization period, hatchlings were anesthetized in 2% ethanol/FSIO for 5 minutes before fixation in 4% paraformaldehyde at 4°C for 48 hours. Following fixation, hatchlings were washed four times with 1× marine phosphate-buffered saline (50 mM sodium phosphate, 0.45 M NaCl [pH 7.4]) and dissected, and imaged with a Zeiss Axio Zoom v.16 large-field 561 fluorescent stereo microscope. Aggregate sizes were determined using the aggregate area tool, and statistical analysis was conducted using the Mann-Whitney test on GraphPad Prism.

## Data Availability

Transcriptomic data were deposited in the NCBI GEO under Series GSE237189 (accession numbers GSM7596716–GSM7596730).

## SUPPLEMENTAL MATERIAL

Supplemental Figures msystems.00815-23-s0001.pdf Figures S1 to S3.

Supplemental Tables msystems.00815-23-s0002.xlsx Tables S1 to S6.

ASM does not own the copyrights to Supplemental Material that may be linked to, or accessed through, an article. The authors have granted ASM a non-exclusive, world-wide license to publish the Supplemental Material files. Please contact the corresponding author directly for reuse.

## References

[1] Vásquez A, Forsgren E, Fries I, Paxton RJ, Flaberg E, Szekely L, Olofsson TC. 2012. Symbionts as major modulators of insect health: lactic acid bacteria and honeybees. PLoS One 7:e33188. doi:10.1371/journal.pone.0033188 22427985 PMC3299755

[2] Pudlo NA, Pereira GV, Parnami J, Cid M, Markert S, Tingley JP, Unfried F, Ali A, Varghese NJ, Kim KS, Campbell A, Urs K, Xiao Y, Adams R, Martin D, Bolam DN, Becher D, Eloe-Fadrosh EA, Schmidt TM, Abbott DW, Schweder T, Hehemann JH, Martens EC. 2022. Diverse events have transferred genes for edible seaweed digestion from marine to human gut bacteria. Cell Host Microbe 30:314–328. doi:10.1016/j.chom.2022.02.001 35240043 PMC9096808

[3] Landmann F. 2019. The Wolbachia endosymbionts. Microbiol Spectr 7. doi:10.1128/microbiolspec.BAI-0018-2019 PMC11590423 30953430

[4] Wu H, Xie S, Miao J, Li Y, Wang Z, Wang M, Yu Q. 2020. Lactobacillus reuteri maintains intestinal epithelial regeneration and repairs damaged intestinal mucosa. Gut Microbes 11:997–1014. doi:10.1080/19490976.2020.1734423 32138622 PMC7524370

[5] Schwartzman JA, Koch E, Heath-Heckman EAC, Zhou L, Kremer N, McFall-Ngai MJ, Ruby EG. 2015. The chemistry of negotiation: rhythmic, glycan-driven acidification in a symbiotic conversation. Proc Natl Acad Sci U S A 112:566–571. doi:10.1073/pnas.1418580112 25550509 PMC4299225

[6] Lynch JB, Bennett BD, Merrill BD, Ruby EG, Hryckowian AJ. 2022. Independent host- and bacterium-based determinants protect a model symbiosis from phage predation. Cell Rep 38:110376. doi:10.1016/j.celrep.2022.110376 35172163 PMC8983117

[7] Serbus LR, Casper-Lindley C, Landmann F, Sullivan W. 2008. The genetics and cell biology of Wolbachia-host interactions. Annu Rev Genet 42:683–707. doi:10.1146/annurev.genet.41.110306.130354 18713031

[8] Kaltenpoth M, Winter SA, Kleinhammer A. 2009. Localization and transmission route of Coriobacterium glomerans, the endosymbiont of pyrrhocorid bugs. FEMS Microbiol Ecol 69:373–383. doi:10.1111/j.1574-6941.2009.00722.x 19583787

[9] Herren JK, Paredes JC, Schüpfer F, Lemaitre B. 2013. Vertical transmission of a Drosophila endosymbiont via cooption of the yolk transport and internalization machinery. mBio 4:e00532-12. doi:10.1128/mBio.00532-12 23462112 PMC3585447

[10] Breusing C, Genetti M, Russell SL, Corbett-Detig RB, Beinart RA. 2022. Horizontal transmission enables flexible associations with locally adapted symbiont strains in deep-sea hydrothermal vent symbioses. Proc Natl Acad Sci U S A 119:e2115608119. doi:10.1073/pnas.2115608119 35349333 PMC9168483

[11] Henry LM, Peccoud J, Simon J-C, Hadfield JD, Maiden MJC, Ferrari J, Godfray HCJ. 2013. Horizontally transmitted symbionts and host colonization of ecological niches. Curr Biol 23:1713–1717. doi:10.1016/j.cub.2013.07.029 23993843 PMC3980636

[12] Bright M, Bulgheresi S. 2010. A complex journey: transmission of microbial symbionts. Nat Rev Microbiol 8:218–230. doi:10.1038/nrmicro2262 20157340 PMC2967712

[13] Nyholm SV, Stabb EV, Ruby EG, McFall-Ngai MJ. 2000. Establishment of an animal-bacterial association: recruiting symbiotic vibrios from the environment. Proc Natl Acad Sci U S A 97:10231–10235. doi:10.1073/pnas.97.18.10231 10963683 PMC27829

[14] Ruby EG, McFall-Ngai MJ. 1992. A squid that glows in the night: development of an animal-bacterial mutualism. J Bacteriol 174:4865–4870. doi:10.1128/jb.174.15.4865-4870.1992 1629148 PMC206296

[15] Manara S, Asnicar F, Beghini F, Bazzani D, Cumbo F, Zolfo M, Nigro E, Karcher N, Manghi P, Metzger MI, Pasolli E, Segata N. 2019. Microbial genomes from non-human primate gut metagenomes expand the primate-associated bacterial tree of life with over 1000 novel species. Genome Biol 20:299. doi:10.1186/s13059-019-1923-9 31883524 PMC6935492

[16] Tierney BT, Yang Z, Luber JM, Beaudin M, Wibowo MC, Baek C, Mehlenbacher E, Patel CJ, Kostic AD. 2019. The landscape of genetic content in the gut and oral human microbiome. Cell Host Microbe 26:283–295. doi:10.1016/j.chom.2019.07.008 31415755 PMC6716383

[17] Nyholm SV, McFall-Ngai MJ. 2004. The winnowing: establishing the squid–vibrio symbiosis. Nat Rev Microbiol 2:632–642. doi:10.1038/nrmicro957 15263898

[18] Mcfall-ngai MJ. 1994. Animal-bacterial interactions in the early life history of marine invertebrates: the Euprymna scolopes/vibrio fischeri symbiosis . Am Zool 34:554–561. doi:10.1093/icb/34.4.554

[19] Wei SL, Young RE. 1989. Development of symbiotic bacterial bioluminescence in a nearshore cephalopod, Euprymna scolopes. Mar Biol 103:541–546. doi:10.1007/BF00399586

[20] Olaso C-M, Viliunas J, McFall-Ngai M. 2022. A peptidoglycan-recognition protein orchestrates the first steps of symbiont recruitment in the squid-vibrio symbiosis. Symbiosis 87:31–43. doi:10.1007/s13199-022-00855-y 36177150 PMC9518823

[21] Yip ES, Geszvain K, DeLoney-Marino CR, Visick KL. 2006. The symbiosis regulator RscS controls the syp gene locus, biofilm formation and symbiotic aggregation by Vibrio fischeri. Mol Microbiol 62:1586–1600. doi:10.1111/j.1365-2958.2006.05475.x 17087775 PMC1852533

[22] Shibata S, Yip ES, Quirke KP, Ondrey JM, Visick KL. 2012. Roles of the structural symbiosis polysaccharide (syp) genes in host colonization, biofilm formation, and polysaccharide biosynthesis in Vibrio fischeri. J Bacteriol 194:6736–6747. doi:10.1128/JB.00707-12 23042998 PMC3510638

[23] Mandel MJ, Wollenberg MS, Stabb EV, Visick KL, Ruby EG. 2009. A single regulatory gene is sufficient to alter bacterial host range. Nature 458:215–218. doi:10.1038/nature07660 19182778 PMC2713604

[24] Rotman ER, Bultman KM, Brooks JF, Gyllborg MC, Burgos HL, Wollenberg MS, Mandel MJ, Mullineaux CW. 2019. Natural strain variation reveals diverse biofilm regulation in squid-colonizing Vibrio fischeri. J Bacteriol 201:e00033-19. doi:10.1128/JB.00033-19 30782630 PMC6456852

[25] Yip ES, Grublesky BT, Hussa EA, Visick KL. 2005. A novel, conserved cluster of genes promotes symbiotic colonization and σ^54^-dependent biofilm formation by Vibrio fischeri. Mol Microbiol 57:1485–1498. doi:10.1111/j.1365-2958.2005.04784.x 16102015

[26] Darnell CL, Hussa EA, Visick KL. 2008. The putative hybrid sensor kinase SypF coordinates biofilm formation in Vibrio fischeri by acting upstream of two response regulators. J Bacteriol 190:4941–4950. doi:10.1128/JB.00197-08 18469094 PMC2447025

[27] Norsworthy AN, Visick KL. 2015. Signaling between two interacting sensor kinases promotes biofilms and colonization by a bacterial symbiont. Mol Microbiol 96:233–248. doi:10.1111/mmi.12932 25586643 PMC4465548

[28] Ray VA, Eddy JL, Hussa EA, Misale M, Visick KL. 2013. The syp enhancer sequence plays a key role in transcriptional activation by the σ^54^-dependent response regulator SypG and in biofilm formation and host colonization by Vibrio fischeri. J Bacteriol 195:5402–5412. doi:10.1128/JB.00689-13 24097942 PMC3837961

[29] Morris AR, Visick KL. 2013. Inhibition of SypG-induced biofilms and host colonization by the negative regulator SypE in Vibrio fischeri. PLoS One 8:e60076. doi:10.1371/journal.pone.0060076 23555890 PMC3610818

[30] Brooks JF II, Mandel MJ, O’Toole GA. 2016. The histidine kinase BinK is a negative regulator of biofilm formation and squid colonization. J Bacteriol 198:2596–2607. doi:10.1128/JB.00037-16 26977108 PMC5019070

[31] Ludvik DA, Bultman KM, Mandel MJ. 2021. Hybrid Histidine kinase BinK represses Vibrio fischeri biofilm signaling at multiple developmental stages. J Bacteriol 203:e0015521. doi:10.1128/JB.00155-21 34031036 PMC8407347

[32] Dial CN, Speare L, Sharpe GC, Gifford SM, Septer AN, Visick KL. 2021. Para-aminobenzoic acid, calcium, and c-di-GMP induce formation of cohesive, Syp-polysaccharide-dependent biofilms in Vibrio fischeri. mBio 12:e0203421. doi:10.1128/mBio.02034-21 34607467 PMC8546588

[33] Tischler AH, Lie L, Thompson CM, Visick KL. 2018. Discovery of calcium as a biofilm-promoting signal for Vibrio fischeri reveals new phenotypes and underlying regulatory complexity. J Bacteriol 200:e00016-18. doi:10.1128/JB.00016-18 29463601 PMC6040196

[34] Geszvain K, Visick KL. 2008. The hybrid sensor kinase Rscs integrates positive and negative signals to modulate Biofilm formation in Vibrio Fischeri. J Bacteriol 190:4437–4446. doi:10.1128/JB.00055-08 18441062 PMC2446786

[35] Geszvain K, Visick KL. 2008. Multiple factors contribute to keeping levels of the symbiosis regulator RscS low. FEMS Microbiol Lett 285:33–39. doi:10.1111/j.1574-6968.2008.01209.x 18510559 PMC2575038

[36] Singh P, Brooks JF II, Ray VA, Mandel MJ, Visick KL, Spormann AM. 2015. CysK plays a role in biofilm formation and colonization by Vibrio fischeri. Appl Environ Microbiol 81:5223–5234. doi:10.1128/AEM.00157-15 26025891 PMC4495211

[37] Thompson CM, Tischler AH, Tarnowski DA, Mandel MJ, Visick KL. 2019. Nitric oxide inhibits biofilm formation by Vibrio fischeri via the nitric oxide sensor HnoX. Mol Microbiol 111:187–203. doi:10.1111/mmi.14147 30299554 PMC6392066

[38] Ray VA, Driks A, Visick KL. 2015. Identification of a novel matrix protein that promotes biofilm maturation in Vibrio fischeri. J Bacteriol 197:518–528. doi:10.1128/JB.02292-14 25404700 PMC4285995

[39] Wagner GP, Kin K, Lynch VJ. 2012. Measurement of mRNA abundance using RNA-seq data: RPKM measure is inconsistent among samples. Theory Biosci 131:281–285. doi:10.1007/s12064-012-0162-3 22872506

[40] Bennett BD, Essock-Burns T, Ruby EG. 2020. HbtR, a heterofunctional homolog of the virulence regulator TcpP, facilitates the transition between symbiotic and planktonic lifestyles in Vibrio fischeri. mBio 11:e01624-20. doi:10.1128/mBio.01624-20 32873761 PMC7468203

[41] Isenberg RY, Christensen DG, Visick KL, Mandel MJ. 2022. High levels of cyclic diguanylate interfere with beneficial bacterial colonization. mBio 13:e0167122. doi:10.1128/mbio.01671-22 35916402 PMC9426504

[42] Wang X, Chapman MR. 2008. Sequence determinants of bacterial amyloid formation. J Mol Biol 380:570–580. doi:10.1016/j.jmb.2008.05.019 18565345 PMC2478699

[43] Evans ML, Chapman MR. 2014. Curli biogenesis: order out of disorder. Biochim Biophys Acta 1843:1551–1558. doi:10.1016/j.bbamcr.2013.09.010 24080089 PMC4243835

[44] Austin JW, Sanders G, Kay WW, Collinson SK. 1998. Thin aggregative fimbriae enhance Salmonella enteritidis biofilm formation. FEMS Microbiol Lett 162:295–301. doi:10.1111/j.1574-6968.1998.tb13012.x 9627964

[45] Hu L. 2018. Prevalence of curli genes among Cronobacter species and their roles in biofilm formation and cell-cell aggregation. Int J Food Microbiol 265:65–73. doi:10.1016/j.ijfoodmicro.2017.10.031 29128733

[46] Carter MQ, Louie JW, Feng D, Zhong W, Brandl MT. 2016. Curli fimbriae are conditionally required in Escherichia coli O157:H7 for initial attachment and biofilm formation. Food Microbiol 57:81–89. doi:10.1016/j.fm.2016.01.006 27052705

[47] Prigent-Combaret C, Prensier G, Le Thi TT, Vidal O, Lejeune P, Dorel C. 2000. Developmental pathway for biofilm formation in curli-producing Escherichia coli strains: role of flagella, curli and colanic acid. Environ Microbiol 2:450–464. doi:10.1046/j.1462-2920.2000.00128.x 11234933

[48] Reichhardt C, Jacobson AN, Maher MC, Uang J, McCrate OA, Eckart M, Cegelski L. 2015. Congo red interactions with curli-producing E. coli and native curli amyloid fibers. PLoS One 10:e0140388. doi:10.1371/journal.pone.0140388 26485271 PMC4618944

[49] Jeffries J, Thongsomboon W, Visser JA, Enriquez K, Yager D, Cegelski L. 2021. Variation in the ratio of curli and phosphoethanolamine cellulose associated with biofilm architecture and properties. Biopolymers 112:e23395. doi:10.1002/bip.23395 32894594

[50] Jonas K, Tomenius H, Kader A, Normark S, Römling U, Belova LM, Melefors O. 2007. Roles of curli, cellulose and BapA in Salmonella biofilm morphology studied by atomic force microscopy. BMC Microbiol 7:70. doi:10.1186/1471-2180-7-70 17650335 PMC1949822

[51] Brennan CA, Mandel MJ, Gyllborg MC, Thomasgard KA, Ruby EG. 2013. Genetic determinants of swimming motility in the squid light-organ symbiont Vibrio fischeri. Microbiologyopen 2:576–594. doi:10.1002/mbo3.96 23907990 PMC3948606

[52] Shrestha P, Razvi A, Fung BL, Eichinger SJ, Visick KL. 2022. Mutational analysis of Vibrio fischeri c-di-GMP-modulating genes reveals complex regulation of motility. J Bacteriol 204:e0010922. doi:10.1128/jb.00109-22 35758751 PMC9295575

[53] Zamorano-Sánchez D, Xian W, Lee CK, Salinas M, Thongsomboon W, Cegelski L, Wong GCL, Yildiz FH. 2019. Functional specialization in Vibrio cholerae diguanylate cyclases: distinct modes of motility suppression and C-di-GMP production. mBio 10:e00670-19. doi:10.1128/mBio.00670-19 31015332 PMC6479008

[54] Tischler AH, Vanek ME, Peterson N, Visick KL. 2021. Calcium-responsive diguanylate cyclase CasA drives cellulose-dependent biofilm formation and inhibits motility in Vibrio fischeri. mBio 12:e0257321. doi:10.1128/mBio.02573-21 34749532 PMC8576532

[55] Hussa EA, Darnell CL, Visick KL. 2008. RscS functions upstream of SypG to control the syp locus and biofilm formation in Vibrio fischeri. J Bacteriol 190:4576–4583. doi:10.1128/JB.00130-08 18441059 PMC2446822

[56] Zorraquino V, García B, Latasa C, Echeverz M, Toledo-Arana A, Valle J, Lasa I, Solano C. 2013. Coordinated cyclic-di-GMP repression of Salmonella motility through YcgR and cellulose. J Bacteriol 195:417–428. doi:10.1128/JB.01789-12 23161026 PMC3554008

[57] Beyhan S, Tischler AD, Camilli A, Yildiz FH. 2006. Transcriptome and phenotypic responses of Vibrio cholerae to increased cyclic di-GMP level. J Bacteriol 188:3600–3613. doi:10.1128/JB.188.10.3600-3613.2006 16672614 PMC1482859

[58] Saldaña Z, Xicohtencatl-Cortes J, Avelino F, Phillips AD, Kaper JB, Puente JL, Girón JA. 2009. Synergistic role of curli and cellulose in cell adherence and biofilm formation of attaching and effacing Escherichia coli and identification of Fis as a negative regulator of curli. Environ Microbiol 11:992–1006. doi:10.1111/j.1462-2920.2008.01824.x 19187284 PMC2672964

[59] Oh YJ, Hubauer-Brenner M, Gruber HJ, Cui Y, Traxler L, Siligan C, Park S, Hinterdorfer P. 2016. Curli mediate bacterial adhesion to fibronectin via tensile multiple bonds. Sci Rep 6:33909. doi:10.1038/srep33909 27652888 PMC5031991

[60] Hollenbeck EC, Antonoplis A, Chai C, Thongsomboon W, Fuller GG, Cegelski L. 2018. Phosphoethanolamine cellulose enhances curli-mediated adhesion of uropathogenic Escherichia coli to bladder epithelial cells. Proc Natl Acad Sci U S A 115:10106–10111. doi:10.1073/pnas.1801564115 30232265 PMC6176564

[61] Vidakovic L, Singh PK, Hartmann R, Nadell CD, Drescher K. 2018. Dynamic biofilm architecture confers individual and collective mechanisms of viral protection. Nat Microbiol 3:26–31. doi:10.1038/s41564-017-0050-1 29085075 PMC5739289

[62] Proctor LM, Fuhrman JA. 1990. Viral mortality of marine bacteria and cyanobacteria. Nature 343:60–62. doi:10.1038/343060a0

[63] Huq A, Small EB, West PA, Huq MI, Rahman R, Colwell RR. 1983. Ecological relationships between Vibrio cholerae and planktonic crustacean copepods. Appl Environ Microbiol 45:275–283. doi:10.1128/aem.45.1.275-283.1983 6337551 PMC242265

[64] Turner JW, Malayil L, Guadagnoli D, Cole D, Lipp EK. 2014. Detection of Vibrio parahaemolyticus, Vibrio vulnificus and Vibrio cholerae with respect to seasonal fluctuations in temperature and plankton abundance. Environ Microbiol 16:1019–1028. doi:10.1111/1462-2920.12246 24024909

[65] Hartwick MA, Berenson A, Whistler CA, Naumova EN, Jones SH. 2021. The seasonal microbial ecology of plankton and plankton-associated Vibrio parahaemolyticus in the northeast United States. Appl Environ Microbiol 87:e0297320. doi:10.1128/AEM.02973-20 33990304 PMC8276809

[66] Hussa EA, O’Shea TM, Darnell CL, Ruby EG, Visick KL. 2007. Two-component response regulators of Vibrio Fischeri: Identification, Mutagenesis, and characterization. J Bacteriol 189:5825–5838. doi:10.1128/JB.00242-07 17586650 PMC1952042

[67] Hsieh M-L, Kiel N, Jenkins LMM, Ng W-L, Knipling L, Waters CM, Hinton DM. 2022. The Vibrio cholerae master regulator for the activation of biofilm biogenesis genes, VpsR, senses both cyclic di-GMP and phosphate. Nucleic Acids Res 50:4484–4499. doi:10.1093/nar/gkac253 35438787 PMC9071405

[68] Zamorano-Sánchez D, Fong JCN, Kilic S, Erill I, Yildiz FH. 2015. Identification and characterization of VpsR and VpsT binding sites in Vibrio cholerae. J Bacteriol 197:1221–1235. doi:10.1128/JB.02439-14 25622616 PMC4352665

[69] Hwang S-H, Im H, Choi SH. 2021. A master regulator BrpR coordinates the expression of multiple loci for robust biofilm and rugose colony development in Vibrio vulnificus Front Microbiol 12:679854. doi:10.3389/fmicb.2021.679854 34248894 PMC8268162

[70] Stabb EV, Ruby EG. 2002. RP4-based plasmids for conjugation between Escherichia coli and members of the Vibrionaceae. Methods Enzymol 358:413–426. doi:10.1016/s0076-6879(02)58106-4 12474404

[71] Boettcher KJ, Ruby EG. 1990. Depressed light emission by symbiotic Vibrio fischeri of the sepiolid squid euprymna scolopes. J Bacteriol 172:3701–3706. doi:10.1128/jb.172.7.3701-3706.1990 2163384 PMC213346

[72] Mandel MJ, Stabb EV, Ruby EG. 2008. Comparative genomics-based investigation of resequencing targets in Vibrio fischeri: focus on point miscalls and artefactual expansions. BMC Genomics 9:138. doi:10.1186/1471-2164-9-138 18366731 PMC2330054

[73] Brooks JF II, Gyllborg MC, Cronin DC, Quillin SJ, Mallama CA, Foxall R, Whistler C, Goodman AL, Mandel MJ. 2014. Global discovery of colonization determinants in the squid symbiont Vibrio fischeri . Proc Natl Acad Sci U.S.A 111:17284–17289. doi:10.1073/pnas.1415957111 25404340 PMC4260577

[74] Bao Y, Lies DP, Fu H, Roberts GP. 1991. An improved Tn7-based system for the single-copy insertion of cloned genes into chromosomes of gram-negative bacteria. Gene 109:167–168. doi:10.1016/0378-1119(91)90604-a 1661697

[75] McCann J, Stabb EV, Millikan DS, Ruby EG. 2003. Population dynamics of Vibrio fischeri during infection of Euprymna scolopes. Appl Environ Microbiol 69:5928–5934. doi:10.1128/AEM.69.10.5928-5934.2003 14532046 PMC201191

[76] Visick KL, Hodge-Hanson KM, Tischler AH, Bennett AK, Mastrodomenico V. 2018. Tools for rapid genetic engineering of Vibrio fischeri. Appl Environ Microbiol 84:e00850-18. doi:10.1128/AEM.00850-18 29776924 PMC6029082

[77] Dunn AK, Millikan DS, Adin DM, Bose JL, Stabb EV. 2006. New rfp- and pES213-derived tools for analyzing symbiotic Vibrio fischeri reveal patterns of infection and lux expression in situ. Appl Environ Microbiol 72:802–810. doi:10.1128/AEM.72.1.802-810.2006 16391121 PMC1352280

[78] Miyashiro T, Wollenberg MS, Cao X, Oehlert D, Ruby EG. 2010. A single qrr gene is necessary and sufficient for LuxO-mediated regulation in Vibrio fischeri. Mol Microbiol 77:1556–1567. doi:10.1111/j.1365-2958.2010.07309.x 20662783 PMC2947852

[79] Pollack-Berti A, Wollenberg MS, Ruby EG. 2010. Natural transformation of Vibrio fischeri requires tfoX and tfoY. Environ Microbiol 12:2302–2311. doi:10.1111/j.1462-2920.2010.02250.x 21966921 PMC3034104

[80] Chen S, Zhou Y, Chen Y, Gu J. 2018. Fastp: an ultra-fast all-in-one FASTQ preprocessor. Bioinformatics 34:i884–i890. doi:10.1093/bioinformatics/bty560 30423086 PMC6129281

[81] Li H. 2013. Aligning sequence reads, clone sequences and assembly contigs with BWA-MEM arXiv:1303.3997 [q-bioGN]

[82] Anders S, Pyl PT, Huber W. 2015. Htseq—a python framework to work with high-throughput sequencing data. Bioinformatics 31:166–169. doi:10.1093/bioinformatics/btu638 25260700 PMC4287950

[83] Robinson MD, McCarthy DJ, Smyth GK. 2010. edgeR: a bioconductor package for differential expression analysis of digital gene expression data. Bioinformatics 26:139–140. doi:10.1093/bioinformatics/btp616 19910308 PMC2796818

[84] Burgos HL, Burgos EF, Steinberger AJ, Suen G, Mandel MJ. 2020. Multiplexed competition in a synthetic squid light organ microbiome using barcode-tagged gene deletions. mSystems 5:e00846-20. doi:10.1128/mSystems.00846-20 33323415 PMC7771539

[85] Naughton LM, Mandel MJ. 2012. Colonization of Euprymna scolopes squid by Vibrio fischeri. J Vis Exp e3758:e3758. doi:10.3791/3758 PMC3399469 22414870

[86] Mandel MJ, Schaefer AL, Brennan CA, Heath-Heckman EAC, Deloney-Marino CR, McFall-Ngai MJ, Ruby EG. 2012. Squid-derived chitin oligosaccharides are a chemotactic signal during colonization by Vibrio fischeri. Appl Environ Microbiol 78:4620–4626. doi:10.1128/AEM.00377-12 22522684 PMC3370474

