# Supplementary material for: Transcriptional pathways across colony biofilm models in the symbiont Vibrio fischeri
Source: mSystems. 2023 Dec 21;9(1):e00815-23. doi: 10.1128/msystems.00815-23 (PMC10804989; doi:10.1128/msystems.00815-23)
Supplement: Supplemental Figures — Figures S1 to S3. [file msystems.00815-23-s0001.pdf]

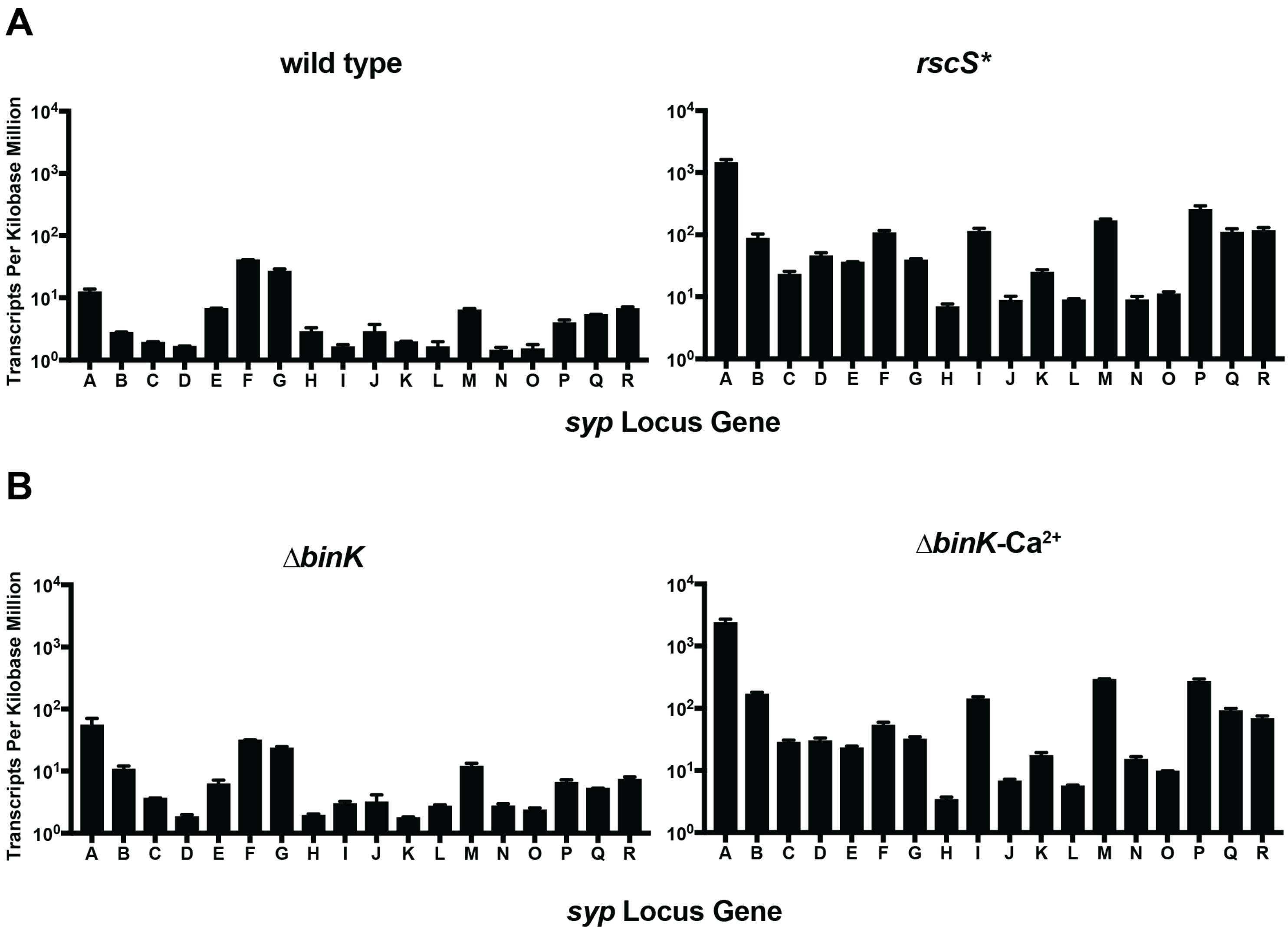

Figure S1. Transcripts Per Kilobase Million (TPM) measurements of RNA-seq data suggest increased basal expression of *sypF* and *sypG*. A. TPM measurements across the *syp* locus for wild type and *rscS** backgrounds. Raw read counts for all genes from the *n* = 3 biological replicates for each background were obtained from HT-Seq output. The read counts for each gene were then divided by each gene's respective length in kilobase pairs to generate reads per kilobase (RPK). These values were summed for each of the replicates and divided by 1,000,000 to generate the scaling factor for each replicate. The RPK for each gene was then divided by its respective replicate scaling factor to generate each genes TPM. This data is contained in Table S5. Once TPMs had been calculated for each replicate, the TPMs for the 18 *syp* locus genes were extracted and plotted in GraphPad Prism. Each bar represents the mean TPM of each gene, and error bars represent the SEM. **B.** TPM measurements across the *syp* locus for Δ*binK* and Δ*binK*-Ca^{2+} backgrounds.

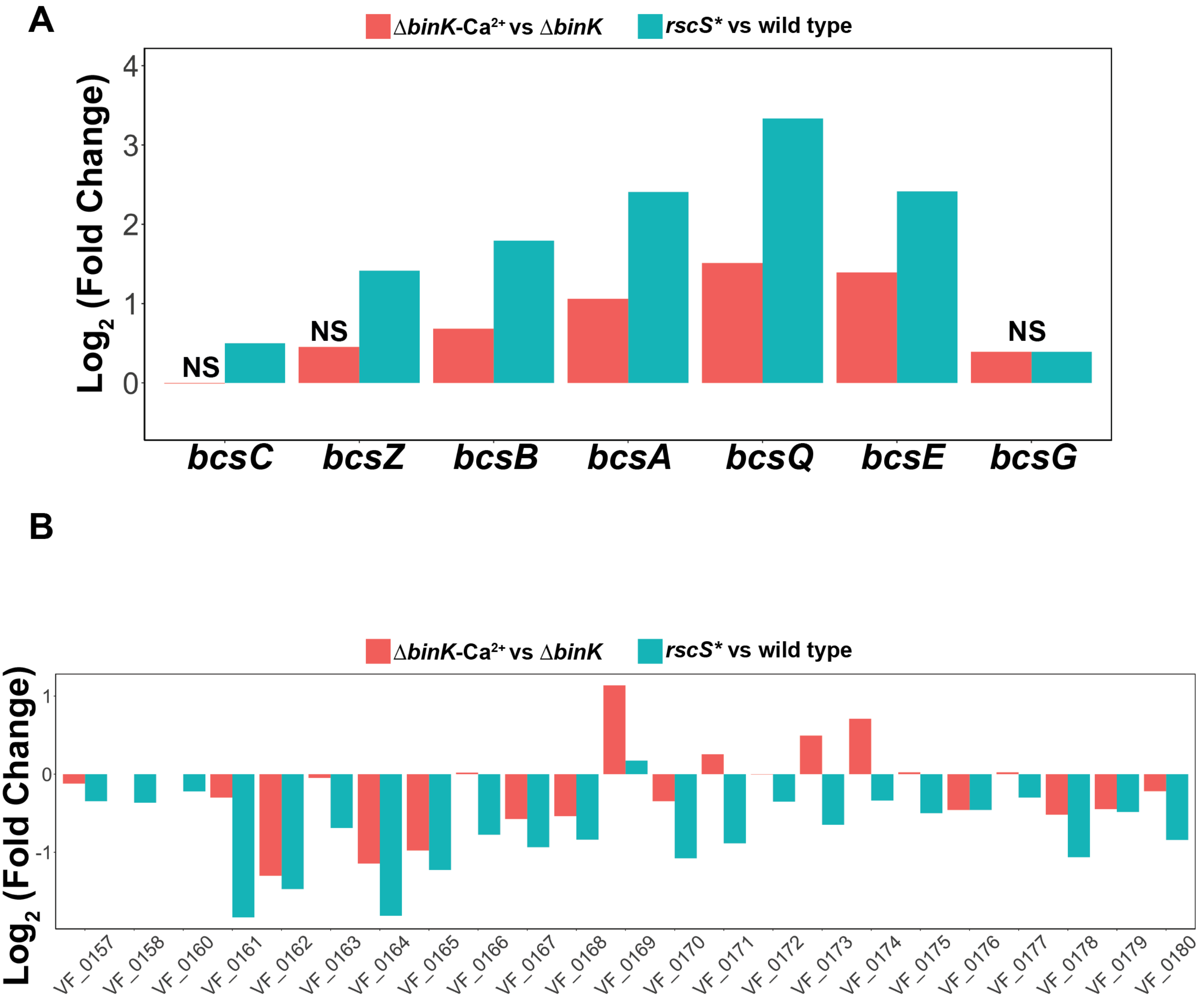

Figure S2. Biofilm induction affects the expression of multiple EPS loci.
A. Cellulose gene (*bcs*) differential expression. Here, the Log_2 Fold Change values for the known cellulose polysaccharide genes are provided from both of the biofilm models. For those genes which were not differentially expressed in one or both analyses, NS (Not Significant) is marked over their respective columns. **B.** *VF_0157-VF_0180* exopolysaccharide locus differential expression. Log_2 Fold Change values for the *VF_0157-VF_0180* exopolysaccharide locus.

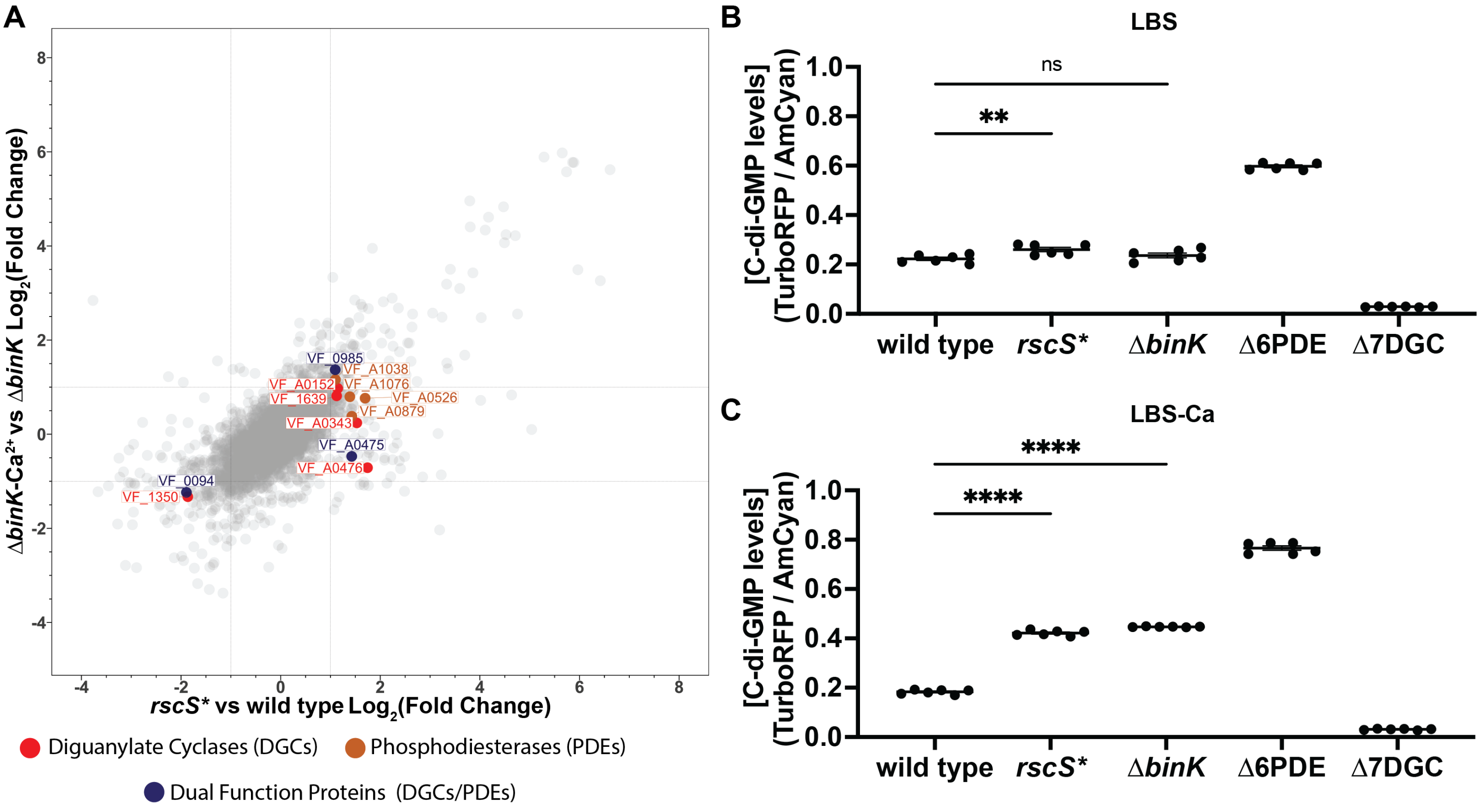

Figure S3. Cyclic-di-GMP levels are impacted by biofilm-induced models

A. Cyclic-di-GMP enzymes plotted on the dual model overlay owing to significance in one or both biofilm models, colors represent function. See Table S2 for detailed statistics for all c-di-GMP enzymes. **B.** Measurement of c-di-GMP levels in colony spots using the pFY4535 reporter on LBS media. Each dot represents one of $n = 6$ biological replicates, each the average of $n = 2$ technical replicates, average bars represent the means of the biological replicates and error bars represent the standard error of the mean. **C.** Measurement of c-di-GMP levels in colony spots using the pFY4535 reporter on LBS-Ca^{2+} media. Each dot represents one of $n = 6$ biological replicates, each the average of $n = 2$ technical replicates, average bars represent the means of the biological replicates and error bars represent the standard error of the mean.
